# Beyond AlphaFold 3: a standardised benchmark of open-source co-folding models for antibody–antigen complexes, from docking accuracy to interaction specificity

**DOI:** 10.1080/19420862.2026.2760737

**Published:** 2026-11-12

**Authors:** Aubin Ramon, Arnav Cheruku, Haowen Zhao, Pietro Sormanni

**Affiliations:** a Department of Chemical Engineering, Imperial College London, London, UK; b Yusuf Hamied Department of Chemistry, University of Cambridge, Cambridge, UK

**Keywords:** Antibody design, antibody docking, antibody engineering, antibody screening, antibody structure prediction, co-folding model, epitope mapping, nanobody, V_H_H

## Abstract

Co-folding models are increasingly used to predict antibody-antigen complexes for epitope mapping, candidate triage and structure-guided de novo design or engineering, yet the latest generation of these models has not been compared under standardized conditions. We benchmark AlphaFold 3, Protenix-v2, ESMFold2, ESMFold2-fast and OpenDDE on 200 test-set complexes (121 Fvs and 79 V_H_Hs) from SAbDab2-Bench, a newly curated dataset, and a reconstructed antibody-antigen subset of FoldBench, Ab-FoldBench. Using standardized inputs, sampling, and interface metrics that isolate the antibody–antigen interface, we compare docking accuracy, inference-time scaling, confidence-based candidate structure selection and sensitivity to interaction perturbations. OpenDDE achieved the highest median DockQ and docking pass rate, followed by Protenix-v2, although their difference was not statistically significant. Both models outperformed AlphaFold 3 on these measures, alongside offering fewer licensing restrictions on model and prediction use. We further find that model confidence was generally effective at identifying accurate predictions but showed model-dependent variations. To probe interaction specificity, which is essential for antibody design, we generated decoys through non-conservative interface mutations, and non-cognate antigen shuffling. Confidence decreased for both perturbations, but more consistently for non-cognate antigens than for interface mutations, with OpenDDE retaining comparatively high confidence for many decoys. Overall, Protenix-v2 combined high docking accuracy with strong rejection of perturbed interfaces, OpenDDE achieved the highest docking accuracy, albeit at the longest runtime, while ESMFold2-fast offered the fastest inference for large-scale triage. These results provide an up-to-date guidance framework for selecting and configuring co-folding models in antibody discovery, design, and engineering.

## 1. Introduction

Deep-learning approaches have transformed protein structure prediction, with AlphaFold2 achieving near-experimental accuracy for many single-chain proteins.^1^ Extending these methods to protein complexes led to co-folding models such as AlphaFold-Multimer.^2^ Modeling quaternary structure is particularly valuable for antibody-antigen complexes, as recovering the correct binding mode and interface yields information that sequence alone cannot provide. Epitope identification supports the inference of mechanisms of action, the prediction of binding competition between candidates, and the rationalization of escape mutations, and has already been pursued directly with co-folding models.^3–5^ Paratope identification separates contact residues, which must be preserved, from peripheral positions that may be mutated to improve developability, for instance by removing aggregation-prone patches, chemical liabilities or immunogenic motifs.^6–8^ Accurate interface geometry is further required for structure-based re-engineering, including *in silico* affinity maturation, complementarity determining region (CDR) grafting, and the tuning of specificity toward or away from related antigens. Beyond characterizing known binders, predicted interfaces provide a triage criterion in discovery campaigns, whether ranking candidates retrieved from antibody libraries^4,9^ or filtering computational *de novo* designs before experimental validation.^10,11^

Antibody-antigen complexes have nonetheless proven harder to model than protein–protein interactions in general, with early benchmarks reporting substantially lower success rates.^12,13^ Two features of the problem contribute. First, paired multiple-sequence alignments (MSAs) carry little inter-chain signal^13^: antibodies and their antigens do not co-evolve across species, since antibody diversity is generated somatically within each individual, so the orthologous pairing that constrains obligate complexes has no counterpart here, and the somatic lineage that produced a given antibody is in most cases unobserved. Second, recognition is mediated by the CDR loops, which are often more conformationally dynamic than typical protein–protein interface elements and can rearrange upon binding, so the bound conformation is not necessarily the one favored by the unbound antibody.^14,15^

AlphaFold 3 (AF3) marked a substantial advance in antibody-antigen co-folding, outperforming AlphaFold-Multimer and showing continued improvements in accuracy with increased inference-time sampling.^16^ However, access to its model parameters remains restricted to noncommercial use and AF3-predicted structures cannot be used to train or finetune other models, motivating the development of open-source alternatives. Among such alternatives, Protenix-v2,^17^ ESMFold2^18^ and OpenDDE^19^ have reported antibody-antigen prediction performance matching or exceeding that of AF3, motivating their inclusion in this study. These models differ not only in architecture, but also in their use of multiple-sequence alignments, structural templates, sampling procedures and confidence metrics.

Despite this rapid progress, comprehensive benchmarking of these models remains limited. FoldBench provides a common low-homology benchmark for biomolecular structure prediction, including 172 antibody–antigen interfaces,^20^ and has been reused by several recent models.^17–19^ Dedicated antibody–antigen assessments have also been reported,^21–24^ showing that success rates increase approximately log-linearly with sampling while internal confidence estimates frequently fail to identify the best model generated for a target,^21^ although these evaluations predated the openly licensed co-folding models considered here. Quite generally, comparisons across studies remain difficult because the benchmark dataset is not always processed or evaluated identically, and inference settings, sampling budgets, and candidate-selection procedures vary between reports. Moreover, confidence metrics are generally inherited from broader protein-complex prediction and are not necessarily evaluated specifically for the antibody-antigen interface. Benchmarking on experimentally established binders measures how accurately the bound complex is recovered, given that binding occurs. It does not test whether confidence estimates reflect interaction specificity, or whether models reject non-binding antibody–antigen pairs, both of which are central to de novo design and to repertoire-scale screening.

Here, we establish a standardized framework for benchmarking antibody-antigen co-folding models. First, we construct SAbDab2-Bench, a curated set of recently deposited antibody-antigen complexes selected to minimize sequence similarity to structures available before the latest model-training cutoff, and combine it with a consistently reconstructed antibody-antigen subset of FoldBench. Then, to extend evaluation beyond known cognate binders, we generate decoy complexes through targeted mutation of key antibody–antigen interface residues and by pairing antibodies with non-cognate shuffled antigens. Using these datasets, we benchmark AF3 against recently released open-source models: Protenix-v2, ESMFold2, ESMFold2-fast and OpenDDE under standardized input preparation, sampling, candidate ranking and evaluation procedures, including role-aware definitions of interface quality and confidence that specifically isolate the antibody–antigen interaction. Beyond comparing docking accuracy, we assess the reliability of interface confidence for selecting accurate predictions, quantify the influence of benchmark composition on accuracy, and test model sensitivity to targeted interface mutations and non-cognate antigens. Together, our results provide a current benchmark of antibody-antigen co-folding models and a reproducible framework for evaluating their structural accuracy, confidence calibration, and sensitivity to interaction specificity.

## 2. Results and discussion

### 2.1. Curated datasets for benchmarking antibody-antigen co-folding models

Reliable evaluation of antibody-antigen co-folding models requires benchmark structures that are distinct from those available during model development. FoldBench^20^ provides an established public benchmark for direct comparison, with a dedicated low-homology antibody-antigen subset on which all model families considered here have been evaluated.^17–19^ However, FoldBench covers a fixed deposition window ending in 2024. The release of SAbDab2,^25^ with its expanded coverage of experimentally determined antibody structures, motivated the construction of a new benchmark comprising more recent structures with low antibody and antigen sequence similarity to the pre-cutoff reference training sets, extending FoldBench.

All model versions evaluated here report a structural-training cutoff of 30 September 2021,^16–19^ allowing complexes released after this date to be treated consistently as post-training structures. From the full SAbDab2, we constructed SAbDab2-Ref, comprising 3,108 structures deposited by 30 September 2021; Ab-FoldBench, comprising 112 complexes from the published FoldBench antibody-antigen subset^20^; and SAbDab2-Bench, comprising 88 complexes all deposited after the 2024 FoldBench deposition window (Figure 1a). Together, the two evaluation sets contained 200 antibody-antigen complexes, comprising 121 Fv- (V_H_–V_L_) and 79 V_H_H-antigen complexes (Figure S1). All three datasets were processed using the same pipeline to ensure consistent structure quality, antibody numbering and antigen assignment (see Methods Section 3 for details of dataset curation).

**Figure 1.**
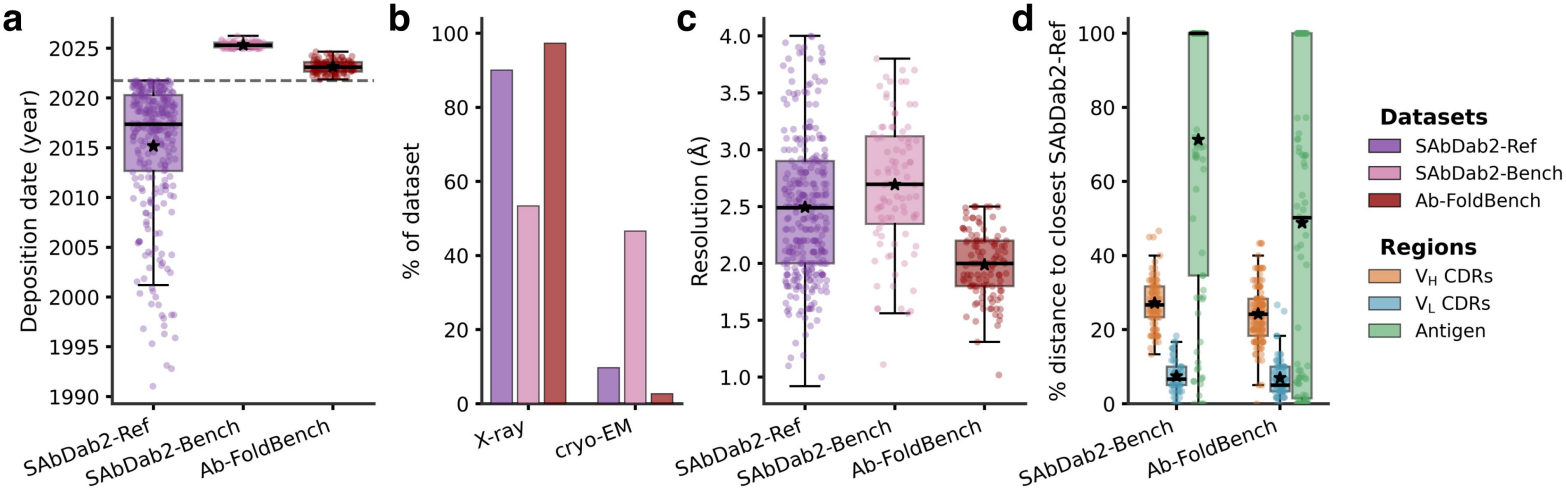
Structural and sequence characteristics of the benchmarking datasets. SAbDab2-ref (purple) comprises SAbDab2 structures deposited on or before the common model-training cutoff of 30 September 2021. SAbDab2-Bench (pink) comprises recently deposited held-out complexes, whereas Ab-FoldBench (burgundy) was reconstructed from the published FoldBench antibody-antigen subset. See methods Section 3 for details of dataset curation. (a) Distributions of PDB deposition dates. The dashed line indicates the common model-training cutoff. (b) Fractions of structures determined by X-ray crystallography and cryogenic electron microscopy (cryo-EM). (c) Distributions of reported experimental resolutions. (d) Sequence distances of the V_H_ CDRs, V_L_ CDRs and antigens from their closest counterparts in SAbDab2-ref. CDR distances are normalised hamming distances across AHo-aligned CDR positions. For each antigen chain, distance is defined as 100 minus the highest BLASTP sequence identity among hits covering more than 80% of the query and is then averaged across chains. Black lines in the box plots indicate medians, and black stars indicate means. Points represent individual complexes and are randomly subsampled where necessary for visualisation. More statistics on the datasets (e.g., antigen and CDR lengths, number of Fvs and V_H_Hs) are available in Figure S1.

As expected from their construction, SAbDab2-Bench contained the most recently deposited structures, whereas Ab-FoldBench occupied an intermediate temporal range (Figure 1). SAbDab2-Bench also showed a marked shift in experimental modality, with 47% of its structures determined by cryogenic electron microscopy (cryo-EM), compared with only 3% of Ab-FoldBench. Ab-FoldBench was biased toward higher-resolution structures, with a median resolution of 2.0 Å, compared with 2.7 Å for SAbDab2-Bench. This difference partly reflects their respective upper resolution thresholds of 2.5 and 4.0 Å. Relative to SAbDab2-Ref, which contains training-set structures for all models we consider, SAbDab2-Bench showed moderately greater sequence novelty in both V_H_ CDRs and antigens than Ab-FoldBench. Thus, SAbDab2-Bench complements Ab-FoldBench by extending evaluation to more recent, experimentally broader, and sequence-diverse complexes.

### 2.2. Standardised inference and evaluation of antibody-antigen co-folding models

The construction of this curated benchmark provided an opportunity to compare recent antibody-antigen co-folding models under a common evaluation framework. We evaluated five models: AlphaFold 3 (AF3),^16^ Protenix-v2,^17^ the ESMFold2 and ESMFold2-fast variants,^18^ and OpenDDE.^19^ AF3 provided an established and highly studied reference baseline, having substantially improved antibody-antigen co-folding accuracy over its predecessor, AlphaFold-Multimer.^2,16^ However, access to AF3 remains restricted: its official weights are available to academic users only for noncommercial research, and further restrictions apply to the modeled structures, which for example cannot be used as synthetic data to train or fine-tune other models. We therefore assessed whether the openly released Protenix-v2, OpenDDE and ESMFold2 models could provide competitive, reproducible alternatives under permissive licenses. To make this comparison as fair as possible, we standardized input preparation, number of seeds and samples, modeled structure ranking and downstream evaluation, while retaining recommended model-specific numbers of recycles and diffusion steps, as these were selected in each original study to optimize the corresponding model performance (Table 1).

**Table 1.** Model configurations and inference settings retained for the primary benchmark.

	AlphaFold 3	Protenix	OpenDDE	ESMFold2	
Model weights	Original	V2	Original	Original	Fast
Training cutoff PDB structures	30 September 2021				
Weights licence	✗ Non-commercial	✔ Apache-2.0		✔ MIT	
MSA	✔ Paired within Fv (V_H_–V_L_) and within Ag chains			✔ Unpaired	✗ Not supported
Template search MSA-based retrieval	✔ Automatic for Ab and Ag			✗ Not supported	
Seeds ∣ Samples	10 ∣ 5				
Recycles ∣ Steps	10 ∣ 200			10 ∣ 68	
Ranking score	$0.8\;{\mathrm{ipTM}}_{max}^{B↔T}+0.2\;\mathrm{pTM}$				

Ab and Ag denote antibody and antigen. Antibody and antigen templates were obtained automatically through standard MSA-based retrieval against a structural database containing only structures predating October 2021. ${\mathrm{ipTM}}_{max}^{B↔T}$ denotes the maximum interface predicted template modelling (TM) score across binder-target chain pairs, where B and T denote binder (Fv or V_H_H) and target (antigen), respectively. See methods Section 3 for further details and Figure S2 for results obtained with extended model configurations.

Central to this standardization is a role-aware definition of interface quality and confidence. Antibody chains were assigned the *binder role* (B) and antigen chains the *target role* (T). The interface-quality metric DockQ was calculated only for the binder–target interface, ignoring any other interface that may be present (e.g., V_H_–V_L_). DockQ combines the fraction of native contacts, the interface root-mean-square deviation (RMSD) and the binder RMSD into a single continuous score in [0,1].^26,27^ Its thresholds were calibrated to reproduce the discrete CAPRI quality classification,^28^ giving incorrect (DockQ ≤0.23), acceptable (0.23 <DockQ ≤ 0.49), medium (0.49 <DockQ ≤0.80) or high quality (DockQ > 0.80) predictions (Figure 2a).

**Figure 2.**
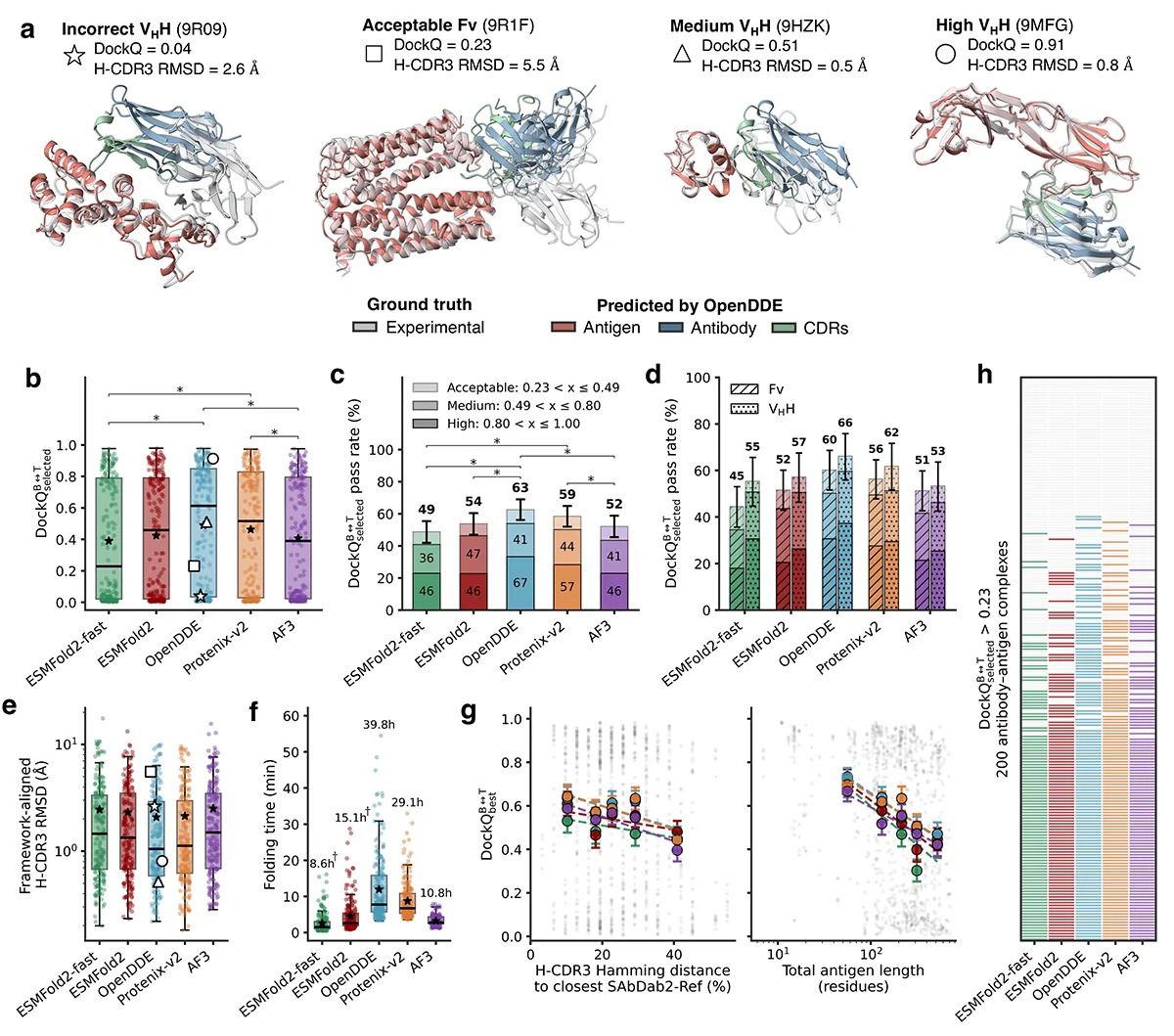
Benchmarking antibody-antigen complex structure prediction. ESMFold2-fast, ESMFold2, OpenDDE, Protenix-v2 and AF3 were evaluated on 200 complexes pooled from SAbDab2-Bench and Ab-FoldBench. Unless otherwise stated, the candidate with the highest common ranking score, R, was selected from 50 predictions per target. Performance on each test set separately is shown in Figure S5. See Table 1 and methods Section 3 for benchmark settings. (a) Experimental structures overlaid with representative OpenDDE predictions from each DockQ class. Experimental structures are grey; predicted antigens, antibody domains and CDRs are salmon, blue and green, respectively. (b) Distributions of ${Do\mathrm{ckQ}}_{se\mathrm{lected}}^{B↔T}.$ Symbols identify the examples in panel a. (c) Overall ${Do\mathrm{ckQ}}_{se\mathrm{lected}}^{B↔T}$ pass rates, partitioned by quality (see legend). (d) Pass rates by antibody format. Panels c-d show mean pass rates across all five-seed subsets drawn from ten seeds, with five samples per seed; error bars show 95% bootstrap confidence intervals over complexes (10,000 resamples), using seed-subset-averaged success rates. Bold labels show total percent pass rates, while labels within bars give category counts. Brackets mark BH-adjusted p<0.05 for median DockQ (b) and pass-rate (c) comparisons in Table 2, using all ten seeds. (e) Distributions of framework-aligned H-CDR3 Cα RMSD. Symbols identify the examples in panel a; the y axis is logarithmic. (f) Distributions of folding time, including MSA and template searches, where applicable. † folding times for the ESMFold2 models were higher than expected may have been affected by the absence of optional acceleration libraries. See Figure S6 for timing benchmarks on different GPU architectures. (g) Highest $\mathrm{Dock}Q^{B↔T}$ across the 50 predictions versus H-CDR3 AHo hamming distance to the closest sequence of the same format (Fv or V_H_H) in SAbDab2-ref (left) and versus total antigen length (right). Coloured points show means across targets within five equal-count bins, with s.e.m. on both axes; grey points show individual complexes for each model and dashed lines show linear fits, computed in log space for the antigen size. (h) Per-complex success at ${Do\mathrm{ckQ}}_{se\mathrm{lected}}^{B↔T}>0.23;$ coloured and grey cells indicate successful and unsuccessful predictions, respectively. Black lines in the box plots indicate medians, and black stars indicate means.

**Table 2.** Pairwise comparison of co-folding model performance.

	OpenDDE	Protenix-v2	AlphaFold 3	ESMFold2	ESMFold2 fast
OpenDDE	*0.61 ∣ 64.0%*	0.374	0.006*	0.006*	<0.001*
Protenix-v2	0.279	*0.52 ∣ 60.5%*	0.049*	0.089	0.006*
AlphaFold 3	0.035*	0.035*	*0.39 ∣ 53.5%*	1.000	0.374
ESMFold2	0.117	0.378	0.572	*0.46 ∣ 54.0%*	0.308
ESMFold2-fast	<0.001*	0.008*	0.317	0.117	*0.23 ∣ 50.0%*

OpenDDE, Protenix-v2 and AlphaFold 3 use automatic antibody and antigen templates; ESMFold2 variants use no templates. All five models were evaluated on the same 200 complexes, so comparisons are paired. *p*-values were adjusted across the ten pairwise comparisons using the Benjamini–Hochberg procedure, separately for each test. Asterisks indicate adjusted p<0.05. for each model and target, the prediction with the highest common ranking score, R, was selected from the 50 candidates generated across ten seeds and five diffusion samples. The pass rates therefore correspond to the full ten-seed sampling budget rather than to the five-of-ten seed subset means shown in Figure 2c. Diagonal entries (*italic*) report each model’s median ${Do\mathrm{ckQ}}_{se\mathrm{lected}}^{B↔T}$ and pass rate (percentage of targets with ${Do\mathrm{ckQ}}_{se\mathrm{lected}}^{B↔T}>0.23$). Entries above the diagonal report adjusted *p*-values from the exact McNemar test comparing pass rates. Entries below the diagonal report adjusted *p*-values from a paired label-swap permutation test on the difference in median ${Do\mathrm{ckQ}}_{se\mathrm{lected}}^{B↔T},$ corresponding to the statistic shown in Figure 2b. For the permutation test, model labels were randomly exchanged within each target over 20,000 permutations.

Similarly, pairwise values of the interface predicted template modeling score (ipTM) and the interaction prediction score from aligned errors (ipSAE) were restricted to binder-target chain pairs. Conventionally, the default returned maximum pairwise ipTM is calculated without distinguishing binder and target roles. It therefore captures information from all interfaces in the complex, including the V_H_–V_L_ interface, and hence also reflects antibody Fv assembly rather than only antigen-binding confidence. We consequently defined ${\mathrm{ipTM}}_{max}^{B↔T}$ as the maximum ipTM across antibody-antigen chain pairs and ranked candidates consistently using

(1) $$R\;=\;0.8\;{\mathrm{ipTM}}_{max}^{B↔T}+0.2\;\mathrm{pTM}.$$

We then harmonized model inputs wherever supported. The same underlying per-chain MSAs and automatic template-search results were generated once and reused across models. Because several MSA-pairing and template configurations were possible, we evaluated these choices separately (Figure S2). For AF3, pairing MSAs across the complete complex provided no appreciable benefit over pairing chains separately within the antibody and antigen: acceptable-quality DockQ pass rates were 52.3% and 52.2%, respectively, using automatically retrieved templates. This is consistent with the limited inter-chain co-evolutionary information generally available for antibody-antigen complexes.^12^

Automatic template searches may retrieve the antigen itself or only related structures, depending on how well characterized each target was before the template cutoff of the model used. To remove this source of variability across models, we applied a common template cutoff date and, for each template search, excluded templates whose sequence was identical to the query over more than 95% of its length.

We then assessed separately whether explicitly supplying the known antigen structure improved docking beyond what the automatic search retrieved. Providing the antigen in the conformation observed in its cognate antibody-bound structure increased docking pass rates for AF3, Protenix-v2 and OpenDDE, with larger gains in the fraction of high-quality predictions. For Protenix-v2, the mean number of complexes yielding a high-quality prediction rose from 57 to 71 out of 200 (Figure S2). Only the antigen coordinates were supplied, without the antibody or its binding pose. However, the antigen conformation supplied is the one recognized by the cognate antibody, so these gains represent an optimistic estimate of what a template can contribute for a target without a known antibody-bound structure.

To assess how strongly the observed benefits generalized beyond cognate-bound templates, we assembled a subset of 119 complexes for which an alternative antigen structure was available in the Protein Data Bank (PDB), determined either in the apo state or, when no apo structure was available, bound to a partner other than the antibody being evaluated (see Methods Section 3). Structural differences with the cognate-bound antigen were generally modest: the median epitope RMSD after local superposition was 0.78 Å (Figure S3a), consistent with previous analyses reporting typically modest antigen conformational changes upon antibody binding.^29^ On this subset, non-cognate templates produced more modest gains in high-quality predictions. OpenDDE showed no increase, while for Protenix-v2 the increase was 2.4% points relative to the aforementioned automatic template search, compared with 8.4 points for cognate-bound templates (Figure S3b).

These results support supplying an experimentally determined target antigen structure when one is available for prospective antibody modeling, although the benefit depends on the model and template conformation. For the primary benchmark in the main text, we used automatic antibody and antigen templates (excluding exact template subsequences spanning more than 95% of the query) wherever supported, since suitable non-cognate antigen structures were unavailable for part of the dataset. This allowed all models to be compared across the full benchmark without supplying the cognate-bound antigen conformation. MSAs were paired separately within the antibody and antigen; ESMFold2 used unpaired MSAs and ESMFold2-fast used no MSAs, and neither supported structural templates.

We used the original model weights wherever applicable, except for Protenix, for which the updated Protenix-v2 model and weights (v2.ckpt) were evaluated. OpenDDE also provides antibody-antigen-specific weights (abag.ckpt), but these did not improve the overall DockQ pass rate over the standard weights (60% versus 62% respectively; Figure S4). We therefore retained the standard weights for the primary, main-text analysis.

Finally, we assessed run-to-run reproducibility by repeating Protenix-v2 inference three times for five nanobody–antigen complexes, using identical seeds and sample counts (Methods Section 3.9). Under default settings, ipTM and pTM varied across runs in a complex-dependent manner, showing that fixed seeds alone do not guarantee reproducibility (Table S6). Enforcing deterministic PyTorch algorithms eliminated this variation but increased runtime by approximately 20%; deterministic execution was therefore disabled for the primary analysis. Such non-determinism may be a general feature of folding models and may explain the limited reproducibility of benchmarks.

### 2.3. Performance in antibody-antigen complex prediction

Using the inference settings identified above, we evaluated the five models on the pooled set of 200 complexes. Each model generated five diffusion samples for each of ten seeds, corresponding to 10,000 predicted structures per model (50 per complex per model). To compare performance at a five-seed sampling budget, we evaluated all 252 subsets of five seeds, using the highest-ranked prediction by R (i.e., the model-selected structure) from the 25 candidates available in each subset. Reported pass rates were averaged across these subsets, with 95% confidence intervals obtained by bootstrapping complexes. DockQ distributions and per-complex comparisons used the model-selected prediction from all 50 candidates (Figure 2).

OpenDDE achieved the highest overall docking accuracy, with a median ${Do\mathrm{ckQ}}_{se\mathrm{lected}}^{B↔T}$ of 0.61 (Figure 2b). Across the five-of-ten seed subsets shown in panel c, an average of 67 of the 200 complexes (33%) had high-quality predictions (${Do\mathrm{ckQ}}_{se\mathrm{lected}}^{B↔T}>0.8$). Protenix-v2 followed closely, with a median DockQ of 0.52 and an average of 57 high-quality predictions (28%). In comparison, AF3 and ESMFold2 achieved overall pass rates (${Do\mathrm{ckQ}}_{se\mathrm{lected}}^{B↔T}>0.23$) of 52% and 54%, respectively, compared with 59% for Protenix-v2. Overall, ESMFold2-fast showed the weakest performance, with the lowest median DockQ (0.23) and pass rate (49%).

Table 2 reports two paired tests of statistical significance for these performance differences. Neither resolves adjacent models. On median ${Do\mathrm{ckQ}}_{se\mathrm{lected}}^{B↔T},$ which reflects both the acceptable pass rate and the absolute quality of the modelled structures but is sensitive to the bi-modality of the underlying distribution (Figure 2b), OpenDDE separated from AF3 (p =  0.035) and ESMFold2-fast (P<0.001), but not from Protenix-v2 (p =  0.279) or ESMFold2 (p =  0.117). Protenix-v2 likewise separated from AF3 and ESMFold2-fast, but not from ESMFold2. The acceptable pass rate told a consistent story, separating both OpenDDE and Protenix-v2 from AF3 and ESMFold2-fast, and additionally separating OpenDDE from ESMFold2 (p =  0.006), while leaving OpenDDE and Protenix-v2 unresolved (p =  0.374). The remaining pairwise comparisons were not significant. Taken together, the two metrics place OpenDDE at the top of the observed ranking, but do not establish an advantage over Protenix-v2.

Unlike the other models, ESMFold2 models do not use antigen templates. With automatic antigen templates, AF3 performed comparably to ESMFold2 in both pass rates and numbers of high-quality structures. Supplying the cognate-bound antigen structure improved AF3’s performance in the separate template comparison (Figure S2).

Performance was broadly consistent across Ab-FoldBench and SAbDab2-Bench, with the same model ranking retained (Figure S5). Relative to published FoldBench evaluations, AF3 performed slightly better in our reconstruction (53% versus ∼ 48%^17–20^), whereas Protenix-v2 and OpenDDE were modestly lower (60% versus ∼ 65%^17^ and 65% versus ∼ 70%,^19^ respectively); ESMFold2 and ESMFold2-fast were broadly comparable to reported values.^18,19^ These small differences likely reflect dataset reconstruction and evaluation protocol differences, including the exclusion of six complexes with incomplete core antibody coordinates. Importantly, all models were evaluated here using the same preprocessing, sampling and ranking framework, providing a more internally consistent comparison across methods.

Stratification by antibody format revealed a stronger dataset-dependent effect. In Ab-FoldBench, V_H_H pass rates were similar to, or lower than, those for Fvs; for example, OpenDDE achieved 61.6% for V_H_Hs and 67.4% for Fvs. By contrast, V_H_Hs were substantially easier in SAbDab2-Bench, while Fvs appeared harder: OpenDDE achieved 72.1% on V_H_H compared with 51.1% for Fvs. This reversal was observed across all models and explains the higher pooled performance on V_H_Hs (Figure 2d). On the one hand, avoiding the need to resolve the V_H_–V_L_ orientation may make single-domain antibodies intrinsically easier to model, on the other, their typically longer H-CDR3 loop that spans a broader range of kinked or extended conformations may make them harder.^22^ In our benchmark, one possible contributor to this discrepancy is that SAbDab2-Bench and Ab-FoldBench have rather different H-CDR3 length distributions, particularly for Fvs, median lengths of 15 and 13 residues respectively (Figure S1c). Quite generally, this dataset-dependent performance discrepancy illustrates the limitations of drawing modality-specific conclusions from either test set alone, particularly given the small number of complexes in each subgroup. It also motivates pooling complementary benchmarks to obtain a larger and more representative evaluation set. Accordingly, although V_H_Hs performed better in the combined analysis, the available data do not provide sufficient evidence that this reflects a general advantage of the V_H_H format.

OpenDDE and Protenix-v2 also produced the most accurate H-CDR3 conformations. Their median H-CDR3 Cα RMSDs, calculated following framework superposition, were 1.05 and 1.13 Å, respectively, compared with 1.50 Å for AF3, 1.34 Å for ESMFold2 and 1.46 Å for ESMFold2-fast (Figure 2e).

OpenDDE provided the highest accuracy but was also the slowest model, requiring 40 hours to generate the 10,000 models (50 for each of the 200 targets), compared with 10.8 hours for AF3 and 8.6 hours for ESMFold2-fast (Figure 2f). ESMFold2 runtimes were slower than previously reported, likely because compatible versions of several optional acceleration libraries were unavailable on our compute system. These end-to-end runtimes also included input-preparation overheads. MSA generation incurred an average amortized cost of 19 s per Fv complex and 10 s per V_H_H complex, while automatic template preparation required an additional 22 and 15 s, respectively. These overheads were avoided by ESMFold2-fast. Overall, OpenDDE offered the highest accuracy, whereas ESMFold2-fast provided the fastest end-to-end workflow.

H-CDR3 sequence novelty showed only a weak relationship with ${Do\mathrm{ckQ}}_{\mathrm{best}}^{B↔T}$ (Figure 2g), with Spearman correlations between −0.07 and −0.17 depending on the model, reaching nominal significance for AF3 (*p* = 0.019) and Protenix-v2 (p =  0.033). Conversely, antigen size was a considerably stronger correlate (Figure 2g), with Spearman correlations between −0.28 and −0.38 ($P<7\times 10^{-5}$). Thus, within the range represented by these benchmarks, the size of the antigen was more strongly associated with docking performance than the sequence novelty of the H-CDR3 loop, which is typically both the hardest loop to model and the most responsible for antigen recognition. This finding is consistent with the simple intuition that a larger antigen has more possible epitopes, thus making the docking problem harder. Docking accuracy was also correlated with experimental method (X-ray vs cryo-EM) and structure resolution. Yet, its correlation with antigen length was even stronger (Figure S7), suggesting that antigen size is the main determinant of the observed docking performance.

The per-complex success patterns revealed limited orthogonality between models (Figure 2h). At least one model produced an acceptable selected prediction for 151 of the 200 complexes (75.5%), compared with 128 complexes (64.0%) for the best individual model, OpenDDE. All five models succeeded on 72 complexes, whereas none succeeded on 49. An oracle combination of models would therefore potentially rescue 23 additional complexes beyond OpenDDE alone, increasing the pass rate from 64.0% to 75.5%. Yet, identifying these cases without access to the ground-truth native structure would require sufficiently reliable confidence estimates.

### 2.4. Evaluating confidence reliability across co-folding models

DockQ provides a direct measure of prediction accuracy but requires a known experimental complex and is therefore unavailable in prospective applications, such as antibody design, candidate screening or epitope mapping, where the antibody-antigen binding mode has not been experimentally determined. We therefore assessed whether the confidence metrics reported by each model could distinguish accurate binding modes and reliably select among the generated candidates.

The role-aware ${\mathrm{ipTM}}_{max}^{B↔T}$ correlated strongly with ${Do\mathrm{ckQ}}_{se\mathrm{lected}}^{B↔T}$ across all targets, with Spearman correlations ranging from 0.77 to 0.82 (Figure 3a). AF3 showed the highest correlations, approximately 0.82, followed by Protenix-v2, ESMFold2-fast, ESMFold2 and OpenDDE. Comparison with alternative confidence metrics (e.g., pLDDT or ipSAE) gave Spearman correlations between 0.71 and 0.81 (**Figure S8**). ${\mathrm{ipTM}}_{max}^{B↔T}$ performed best for OpenDDE and AF3, whereas maximum ipSAE performed marginally better for Protenix-v2 and the two ESMFold2 variants. The differences were generally small, supporting the use of the consistently defined ${\mathrm{ipTM}}_{max}^{B↔T}$ for comparisons between models.

**Figure 3.**
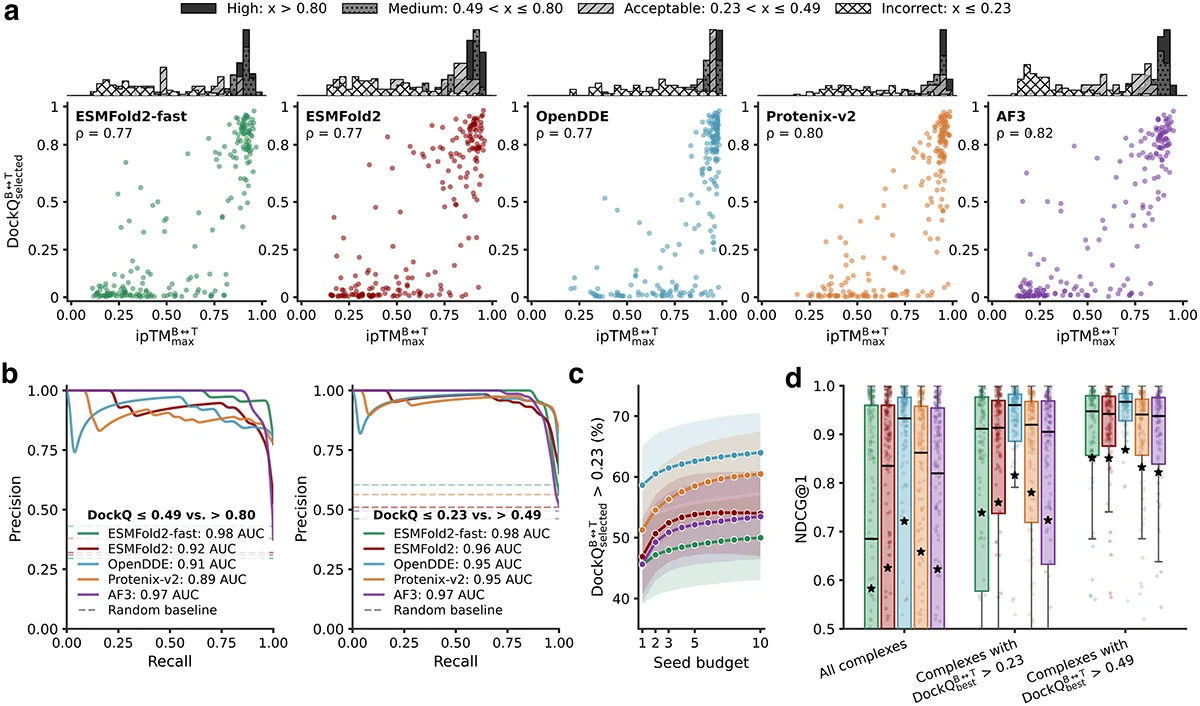
Confidence-based ranking of antibody-antigen complex structure predictions. ESMFold2-fast, ESMFold2, OpenDDE, Protenix-v2, and AF3 were evaluated on 200 complexes pooled from SAbDab2-Bench and Ab-FoldBench. Each model generated five diffusion samples for each of ten seeds. See methods Section 3 for more details. (a) ${\mathrm{IpTM}}_{max}^{B↔T}$ Versus ${Do\mathrm{ckQ}}_{se\mathrm{lected}}^{B↔T}$ for the top-ranked prediction for each target. Marginal histograms show separately normalised maximum-ipTM distributions for the four prediction-quality categories indicated by the greyscale and hatch-pattern legend, where x denotes DockQ. Values within panels give Spearman’s ρ, all with a two-sided P value $<10^{-39}.$ (b) Precision-recall curves distinguishing predictions with ${Do\mathrm{ckQ}}_{se\mathrm{lected}}^{B↔T}\leq 0.49$ from those with ${Do\mathrm{ckQ}}_{se\mathrm{lected}}^{B↔T}>0.80$ (on the left) and ${Do\mathrm{ckQ}}_{se\mathrm{lected}}^{B↔T}\leq 0.23$ from those with ${Do\mathrm{ckQ}}_{se\mathrm{lected}}^{B↔T}>0.49$ (on the right) using ${\mathrm{ipTM}}_{max}^{B↔T}.$ Predictions between the stated thresholds were excluded from each comparison. Legends report the area under the precision–recall curve and dashed lines indicate the corresponding random baselines. Corresponding ROC curves are shown in Figure S9. (c) DockQ pass rate (${Do\mathrm{ckQ}}_{se\mathrm{lected}}^{B↔T}>0.23$) as a function of seed budget. for each budget k, all $\left(\begin{array}{c} 10\\ k \end{array}\right)$ seed subsets were evaluated, with five samples per seed and the prediction with the highest R selected. Points show mean pass rates across targets, and shaded regions indicate 95% bootstrap confidence intervals over complexes (10,000 resamples). (d) Distributions of NDCG@1 for all complexes, complexes with at least one candidate with DockQ>0.23, and complexes with at least one candidate with DockQ>0.49. NDCG@1 is the DockQ of the prediction selected by R divided by the maximum DockQ among the 50 candidates. Black lines in the box plots indicate medians, and black stars indicate means. The y-axis is truncated at 0.5.

We next considered two practically distinct classification tasks (Figure 3b). The first distinguished inaccurate or low-quality from high-quality predictions (${Do\mathrm{ckQ}}_{se\mathrm{lected}}^{B↔T}\leq 0.49$ versus >0.80), representing the accuracy required for atomic-level applications such as identifying specific antibody-antigen contacts, guiding affinity-maturation mutations or redesigning the binding interface. We deliberately excluded medium-quality predictions (0.49 < ${Do\mathrm{ckQ}}_{se\mathrm{lected}}^{B↔T}\leq 0.8$), because misassigning an intermediate case carries little practical cost: such predictions can reasonably be retained or discarded. The errors that matter are those spanning the extremes, namely rejecting a prediction accurate enough to act on, or accepting one whose binding mode is wrong or inaccurate. The values of the area under the precision–recall curve (PR-AUC) ranged from 0.89 for Protenix-v2 to 0.98 for ESMFold2-fast.

The second task distinguished incorrect predictions from medium- or high-quality docking modes (${Do\mathrm{ckQ}}_{se\mathrm{lected}}^{B↔T}\leq 0.23$ versus >0.49), testing whether the model identified the correct epitope and approximate binding pose rather than the precise atomic arrangement of the interface. Confidence-based discrimination was similarly strong, with PR-AUC values between 0.95 and 0.98. Notably, ESMFold2-fast achieved the highest PR-AUC (0.98; random baseline, 0.46), despite having the lowest docking pass rate (50.0%, compared with 64.0% for OpenDDE, whose PR-AUC was 0.95 against a random baseline of 0.60). Receiver operating characteristic (ROC) analysis corroborated these findings, with ESMFold2-fast achieving the highest observed ROC-AUC in both tasks (0.99 and 0.97, respectively; Figure S9).

The optimal ipTM thresholds differed substantially between models (Table 3), particularly for docking success, where they ranged from 0.73 for AF3 to 0.86 for OpenDDE. Across models, the optimal thresholds for identifying docking success were consistently lower than those required for high-quality predictions. For example, for Protenix-v2, the optimal ipTM threshold decreased from 0.91 for high-quality prediction to 0.79 for docking success. At these thresholds, false discovery rates ranged from 4.1–17.9% for high-accuracy structure prediction and 4.8–6.7% for docking success, while false negative rates ranged from 1.5–6.1% and 2.9–12.5%, respectively. Table 3 also reports bootstrap confidence intervals to indicate uncertainty in the cutoffs and corresponding error rates.

**Table 3.** Model-specific optimal ${\mathrm{ipTM}}_{max}^{B↔T}$ cutoffs for high-accuracy structure prediction and docking success.

	AlphaFold 3	Protenix-v2	OpenDDE	ESMFold2	ESMFold2 fast
High-accuracy structure prediction: ${Do\mathrm{ckQ}}_{se\mathrm{lected}}^{B↔T}\leq 0.49$ vs. ${Do\mathrm{ckQ}}_{se\mathrm{lected}}^{B↔T}>0.80$					
Best ${\mathrm{ipTM}}_{max}^{B↔T}$ cutoff	0.78±0.07	0.91±0.05	0.90±0.03	0.79±0.10	0.83±0.04
False discovery rate $\mathrm{FDR}=\frac{\mathrm{FP}}{\mathrm{TP}+\mathrm{FP}}$	14.5±12.9%	13.6±9.7%	17.3±8.4%	17.9±12.6%	4.1±6.3%
False negative rate $\mathrm{FNR}=\frac{\mathrm{FN}}{\mathrm{TP}+\mathrm{FN}}$	4.1±7.6%	5.0±5.1%	1.5±3.8%	6.1±7.4%	2.1±3.9%
Docking success (epitope mapping): ${Do\mathrm{ckQ}}_{se\mathrm{lected}}^{B↔T}\leq 0.23$ vs. ${Do\mathrm{ckQ}}_{se\mathrm{lected}}^{B↔T}>0.49$					
Best ${\mathrm{ipTM}}_{max}^{B↔T}$ cutoff	0.73±0.10	0.79±0.05	0.86±0.05	0.74±0.09	0.75±0.06
False discovery rate $\mathrm{FDR}=\frac{\mathrm{FP}}{\mathrm{TP}+\mathrm{FP}}$	4.8±6.0%	6.6±4.7%	6.2±4.6%	6.7±6.8%	4.8±5.1%
False negative rate $\mathrm{FNR}=\frac{\mathrm{FN}}{\mathrm{TP}+\mathrm{FN}}$	12.1±7.6%	2.9±4.6%	4.5±5.4%	12.5±7.4%	7.0±6.5%

AlphaFold 3, Protenix-v2 and OpenDDE use automatic antibody and antigen templates; ESMFold2 variants use no templates. for each target, the prediction with the highest ranking score, $R=0.8\;{\mathrm{ipTM}}_{max}^{B↔T}+0.2\;\mathrm{pTM},$ was selected from the 50 candidates generated across all seeds and diffusion samples. Cutoffs maximise Youden’s J for two classification tasks. The high-accuracy structure prediction task distinguishes predictions with ${Do\mathrm{ckQ}}_{se\mathrm{lected}}^{B↔T}>0.80$ from those with ${Do\mathrm{ckQ}}_{se\mathrm{lected}}^{B↔T}\leq 0.49.$ the docking-success task distinguishes predictions with ${Do\mathrm{ckQ}}_{se\mathrm{lected}}^{B↔T}>0.49$ from those with ${Do\mathrm{ckQ}}_{se\mathrm{lected}}^{B↔T}\leq 0.23.$ Predictions between the thresholds of each task were excluded. The false discovery rate is $\mathrm{FDR}=\mathrm{FP}/\left(\mathrm{TP}+\mathrm{FP}\right),$ and the false negative rate is $\mathrm{FNR}=\mathrm{FN}/\left(\mathrm{TP}+\mathrm{FN}\right),$ where TP, FP and FN denote true positives, false positives and false negatives, respectively. Values show estimates ± approximate 95% confidence interval half-widths, calculated from 10,000 resamples of complexes. The optimal cutoff was re-estimated in each resample, and FDR and FNR were evaluated at that cutoff within the same resample. Separate tables for Fvs and V_H_Hs are available in table S1.

These cutoffs illustrate model- and task-dependent calibration and should be regarded as empirical starting points. Because each cutoff was selected and evaluated on the same 200 complexes, the reported false discovery and false negative rates are in-sample estimates and are likely to be optimistic for new complexes. The bootstrap intervals in Table 3 capture variability arising from threshold selection, but not this optimism. Besides, some applications may benefit from a different balance of FDR and FNR, which can be achieved by increasing or lowering the ipTM cutoffs. For example, in *de novo* design, provided that enough candidates can be generated, a false positive is more problematic than a false negative, as the former is a failed design leading to wasted experimental resources, while the latter is a missed opportunity (in other words, precision matters more than recall). Conversely, in the screening of sequenced antibody repertoires one may wish to tolerate more false positives to avoid ruling out useful functional antibodies (recall matters more than precision). Quite generally, users may wish to recalibrate these cutoffs on representative labeled data for their application and evaluate their performance on held-out complexes.

We repeated this analysis separately for Fv and V_H_H complexes (Table S1). The optimal thresholds differed between formats, by up to 0.10 ipTM units for the docking-success task, but applying the pooled cutoffs from Table 3 changed some error rates appreciably. For the high-accuracy task, FDR increased from 0.0% to 18.2% for AF3 on Fv and from 11.1% to 17.2% for Protenix-v2 on V_H_H. For the docking-success task, ESMFold2’s FNR on Fv increased from 3.6% to 10.9%. Given that each stratified comparison contains few complexes per class, the pooled model-specific thresholds remain the better default for either format.

The preceding analysis assessed how well confidence metrics classify predictions in absolute terms, against fixed quality thresholds. We next asked a different question: given the candidates generated across seeds and diffusion samples for one target, how reliable are confidence scores at identifying the best one? Ranking within a target and classifying across targets are distinct capabilities, and a model may be strong at one and weak at the other.

Increasing the sampling budget (i.e., the number of seeds) improved confidence-based selection, although the gains progressively decreased (Figure 3c). This trade-off is particularly relevant to high-throughput applications, including structure-guided prioritization of antibody-library hits^5^ and screening of *de novo* antibody designs,^10^ where additional sampling per candidate comes at the expense of evaluating more candidates. Most of the improvement in confidence-based selection was achieved within the first five seeds. Increasing the budget from 5 to 10 seeds increased the DockQ pass rate only from 62.6% to 64.0% for OpenDDE, 58.5% to 60.5% for Protenix-v2, 52.2% to 53.5% for AF3 and 48.8% to 50.0% for ESMFold2-fast. ESMFold2 had effectively plateaued by five seeds. Thus, doubling the sampling cost beyond five seeds provided at most a 2.0-percentage-point improvement in pass rate, suggesting limited benefit from additional sampling in high-throughput settings.

To quantify how effectively each model’s confidence score identifies the best of the 50 candidates generated for a target, we used the normalized discounted cumulative gain at rank one (NDCG@1), which is simply the DockQ of the top-ranked prediction (i.e., of the selected candidate) divided by the highest DockQ among all candidates. A value of one denotes perfect selection. Median NDCG@1 ranged from 0.69 for ESMFold2-fast to 0.93 for OpenDDE and increased to 0.91–0.96 in the more relevant case in which at least one acceptable candidate was available (Figure 3d). Thus, ranking reliability varied between models but was generally high when at least one useful candidate had been generated. However, the distribution of NDCG@1 values across targets was long-tailed, with some complexes showing values < 0.6 for all models, denoting the presence of high-quality candidates missed by the confidence ranking.

### 2.5. Sensitivity to interface mutations and non-cognate antigens

The preceding analyses showed that confidence scores such as ${\mathrm{ipTM}}_{max}^{B↔T}$ are reasonably good at distinguishing accurate from inaccurate structural predictions for antibodies and antigens known to interact with each other. However, this does not establish whether the models can recognise pairs that are unlikely to bind, a key requirement for screening antibody candidates, including for *de novo* designs, where a confidently predicted interface may still represent an experimentally unsuccessful interaction. More broadly, deep-learning models can remain overconfident on incorrect, out-of-distribution examples,^30,31^ and protein-complex predictors likewise have been shown not to perfectly discriminate interacting from non-interacting pairs.^32^ We therefore examined whether perturbing a known interface or replacing the cognate antigen reduces predicted confidence in antibody–antigen complexes.

We generated two sets of decoys from the 88 complexes in SAbDab2-Bench (Figure 4a, e). First, we substituted the top 2, 3 or 5 hotspot interface residues, separately in the antibody and antigen, with chemically dissimilar amino acids (BLOSUM62 ≤−1), generating three variants per condition. Hotspot residues were prioritized by their antibody-antigen side-chain contacts and burial upon binding (Figure 4a, S10). Second, we paired each antibody with five selected non-cognate antigens, selected for low structural similarity to the cognate epitope and dissimilarity between the corresponding cognate antibodies’ CDR sequences (Figure 4e, S11). Non-cognate antigen pairing has previously been explored in nanobody complexes.^24^ Here, we broaden this analysis to include Fvs and benchmark recent open-source co-folding models alongside AF3, while explicitly selecting non-cognate antigens that are structurally dissimilar to the cognate antigen’s epitope. Therefore, both interface mutation and antigen shuffling strategies were designed so that the resulting variants are expected not to bind, or at least to bind with a dissociation constant ($K_{D}$) substantially weaker than that of the WT cognate complex.

**Figure 4.**
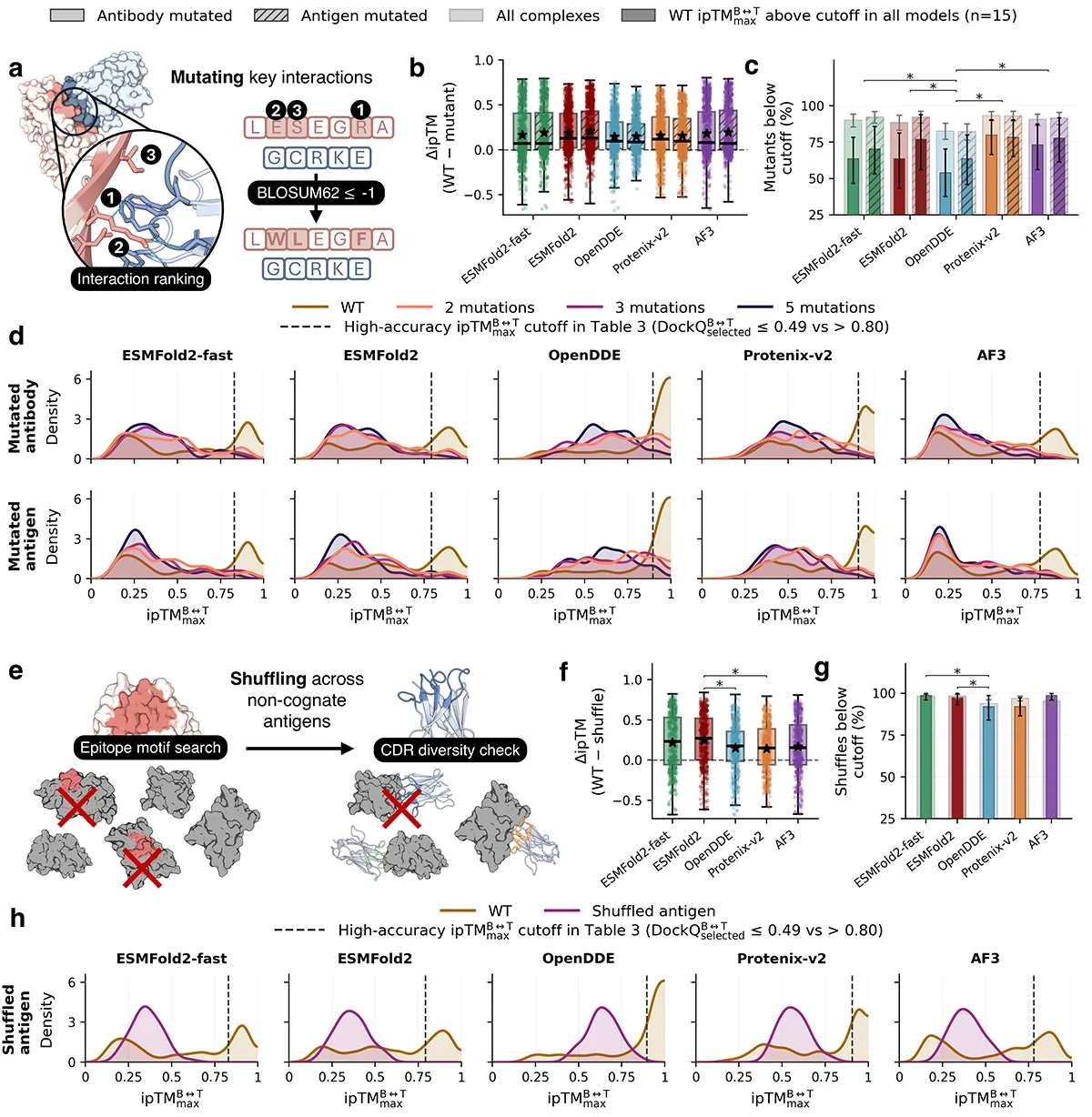
Sensitivity to mutation and antigen shuffling. ESMFold2-fast, ESMFold2, OpenDDE, Protenix-v2 and AF3 were evaluated on 88 complexes from SAbDab2-Bench. for each complex, interface residues of either the antibody or the antigen were substituted to generate three distinct variants at each of three mutation loads (2, 3 and 5 substitutions), and five shuffled-antigen variants were generated. Each wild-type (wt) and variant target was folded with five seeds and five samples per seed, using, where supported, role-scoped MSA pairing and automatic template searches for both partners. From the 25 candidates, the prediction with the highest ranking score, R, was selected. Panels a–d summarise the mutations and panels e–h the antigen shuffling. (a,e) schematic overview of the mutation and antigen-shuffling procedures, described in methods Section 3. (b,f) Distributions of $\Delta {\mathrm{ipTM}=\mathrm{ipTM}}_{max,WT}^{B↔T}{-\mathrm{ipTM}}_{max,va\mathrm{riant}}^{B↔T}.$ Positive values indicate reduced confidence relative to wt. Points represent individual variants; black lines indicate medians and black stars indicate means. (c,g) Percentages of variants below the high-accuracy ipTM cutoffs (higher is better). Light bars include all 88 complexes; narrow dark bars include only complexes whose wt prediction exceeds the high-accuracy ${\mathrm{ipTM}}_{max}^{B↔T}$ cutoff for all models (*n* = 15). Error bars show 95% bootstrap confidence intervals over complexes (10,000 resamples), keeping related variants together. Plain and hatched boxes and bars denote antibody and antigen mutations, respectively. (d,h) ${\mathrm{ipTM}}_{max}^{B↔T}$ distributions for wt and mutated antibody (first mutation row), mutated antigen (second mutation row), and shuffled antigen. Variant scores are averaged within each complex. Colours distinguish mutation counts or wt and shuffled antigen; dashed vertical lines mark the model-specific high-accuracy ipTM cutoffs from Table 3. Brackets mark BH-adjusted p<0.05 as computed in Tables 4 , S2 and S3, using all five seeds on all complexes.

Each cognate and non-cognate pair was evaluated using five seeds and five samples per seed, selecting the candidate with the highest common ranking score, R. Mutations generally reduced confidence: complexes with a mutated interface scored below their WT reference in 69.2–81.1% of cases across models and mutated partners. Median reductions in ${\mathrm{ipTM}}_{max}^{B↔T}$ ranged from 0.07 to 0.13, with no statistically significant pairwise differences between models after multiple-hypothesis testing correction (Figure 4b, Table S3). Thus, all five models showed sensitivity to interface substitutions. However, this does not establish whether confidence alone can reliably filter a set of candidate interacting pairs in the absence of a reference WT complex, as is typically the case in repertoire screening or *de novo* design.

A reduction relative to WT does not necessarily bring a mutant below the confidence threshold used to prioritize candidates. We therefore applied the model-specific high-accuracy ${\mathrm{ipTM}}_{max}^{B↔T}$ cutoffs from Table 3 to ask how often decoys would be excluded by this filter (Figure 4c). Across all 88 complexes, OpenDDE consistently had the lowest below-cutoff fractions for interface mutants, placing 82.8% of antibody mutants below its cutoff, compared with 93.3% for Protenix-v2. In other words, using OpenDDE with its high-accuracy ${\mathrm{ipTM}}_{max}^{B↔T}$ cutoff would result in 17% of interface-mutated complexes being classified as reliable, high accuracy structures. OpenDDE’s above-cutoff fractions were significantly higher than those of all other models for both mutated partners (Tables 4 , S2). Restricting the analysis to the 15 complexes whose WT ${\mathrm{ipTM}}_{max}^{B↔T}$ exceeded the cutoff in all five models showed a similar trend (dark bars in Figure 4c). Separating the mutation counts further highlighted this difference: with two mutations, 76.1% of OpenDDE mutants fell below its cutoff for both mutated partners, compared with 88.6% of antibody mutants for Protenix-v2, across all 88 complexes (Figure S12). Similar trends were observed for antigen mutants (Figure 4).

Antigen shuffling produced larger median confidence reductions than mutations for every model, ranging from 0.15 to 0.27 (Figure 4f, Table S3). Across all models, 93.9–98.4% of shuffled pairs fell below the high-accuracy cutoffs. OpenDDE had the lowest below-cutoff fraction, differing significantly from both ESMFold2 models, but not from Protenix-v2 or AF3 (Figure 4g, Table 4).

Applying the lower epitope-mapping ${\mathrm{ipTM}}_{max}^{B↔T}$ cutoffs from Table 3 retained more decoys above this threshold (Figure S13). For antibody mutants, 76.6% fell below the docking cutoff for OpenDDE and 80.6% for Protenix-v2, compared with 87.9% for AF3. For shuffled antigens, below-cutoff fractions decreased from 93.9% to 89.5% for OpenDDE and from 97.0% to 87.5% for Protenix-v2 (Figure S13). These results reinforce the importance of task-specific ipTM cutoff selection. For stringent candidate filtering, the high-accuracy cutoffs exclude more of these decoys, but the extent of separation is model-dependent and not perfect.

**Table 4.** Pairwise comparison of model responses to antibody mutations.

	OpenDDE	Protenix-v2	AlphaFold 3	ESMFold2	ESMFold2 fast
OpenDDE	93.9% ∣ *82.8%*	<0.001*	<0.001*	0.009*	0.002*
Protenix-v2	0.056	97.0% ∣ *93.3%*	0.259	0.076	0.259
AlphaFold 3	0.390	0.378	95.5% ∣ *90.8%*	0.460	0.847
ESMFold2	0.003*	0.299	0.056	98.4% ∣ *88.8%*	0.460
ESMFold2 fast	0.003*	0.300	0.056	1.000	98.4% ∣ *90.3%*

All models were evaluated on the same 88 complexes, independently of wt eligibility. The 2, 3 and 5 mutation conditions are pooled, with nine individual mutants per complex. Diagonal entries report the percentages below the model-specific high-accuracy ipTM cutoff from Table 3 in the order shuffled antigen ∣ mutant (in italics). Above-diagonal entries report adjusted *p*-values for differences in mean per-complex antibody-mutant below-cutoff fractions. Below-diagonal entries report adjusted *p*-values for differences in mean per-complex shuffled-antigen below-cutoff fractions. Two-sided paired label-swap tests keep all variants from the same complex together and use 20,000 permutations with a plus-one correction; rate tests use exact enumeration when there are at most 16 nonzero paired differences. Benjamini–Hochberg correction is applied across the ten model pairs separately for each test and table. Asterisks indicate adjusted p<0.05. for each model and target (wt, mutant or shuffle), the prediction with the highest common ranking score, R, is selected from the 25 candidates generated across all five seeds and five diffusion samples. Results therefore correspond to the full five-seed sampling budget, matching Figure 4. Additional statistical comparisons are provided for antigen mutations in Table S2 and for ΔipTM responses to mutations and shuffled antigen in Table S3.

Taken together, these results suggest that the models capture broad partner specificity more reliably than fine-grained interface determinants. Antigen shuffling consistently reduced confidence, whereas local interface mutations produced weaker and more variable effects. This issue was most pronounced for OpenDDE, which showed the strongest docking performance but retained comparatively high confidence for many mutant decoys. Overall, current models appear better at rejecting clearly non-cognate partners than at recognizing sequence changes that disrupt or diminish binding.

### 2.6. Conclusions

This study combines a larger, curated benchmark dataset with a standardized framework for comparing the performance of co-folding models in predicting the structure of antibody-antigen complexes, and the resulting picture translates into concrete guidance for antibody engineering. The benchmark structures, curation pipeline and evaluation code are released in full. Because the earliest SAbDab2-Bench deposition is 5 December 2024, the set remains held out for models trained on earlier data and can be regenerated from a later SAbDab2 snapshot as training cutoffs advance, which also makes it a candidate evaluation target for antibody and nanobody design pipelines with more recent cutoffs.

On accuracy, OpenDDE had the highest median DockQ and pass rate, followed by Protenix-v2. ESMFold2 and AF3 performed similarly, while ESMFold2-fast had the lowest values. However, some of these differences, and notably all differences between ranking-adjacent models, did not reach the threshold for statistical significance after multiple-hypothesis testing correction (Table 2). Besides, performance ranking is not the only consideration. OpenDDE required considerably more inference time than AF3 for the same sampling budget, and, among open-source models, Protenix-v2 offered the better accuracy-per-hour trade-off for large campaigns, while ESMFold2-fast dispenses with MSA generation altogether and suits triage of very large candidate sets where a lower pass rate is acceptable. Licensing reinforces the case for the open-source models, as AF3 weights are restricted to noncommercial academic use and which constrains downstream use of the predicted structures themselves.

In *de novo* antibody design and repertoire screening, the structure of the antigen to be targeted is often already known and can be supplied as a template. Our results support that this approach may be useful: non-cognate antigen structures improved docking performance for AF3 and Protenix-v2, although gains in high-quality predictions were smaller than with cognate-bound templates, while OpenDDE showed no improvement. The benefit therefore depends on the model and the antigen conformation provided. Conversely, pairing MSAs across the complete complex conferred no benefit over pairing within the antibody and within the antigen separately, consistent with the lack of inter-chain co-evolutionary signal between antibody and antigen in available protein-sequence datasets.

Sampling showed diminishing returns over the range tested: five seeds captured most of the achievable pass rate, and doubling the budget added at most two percentage points, so – unless maximizing accuracy is truly critical for the problem at hand – compute is better spent on additional targets than on additional seeds per target. However, sampling up to ten seeds cannot establish an eventual performance plateau, and applications requiring highly accurate interface recovery, such as structure-guided affinity maturation, may still benefit from substantially larger sampling budgets. Confidence estimates were informative but require model-specific calibration, with optimal ${\mathrm{ipTM}}_{max}^{B↔T}$ cutoffs spanning 0.73 to 0.91 across models and shifting between applications; the thresholds in Table 3 provide starting points, and the role-aware definition should be preferred over the default maximum ipTM.

Two questions lie outside the scope of the presented analysis. First, our benchmark identifies antigen size as a determinant of difficulty (Figure 2g) but does not explain the residual variation: 49 of 200 complexes were missed by every model, and H-CDR3 sequence novelty accounted for little of it (Figure 2g). Other determinants reported elsewhere, including H-CDR3 length,^23^ kinked versus extended CDR3 conformations in single-domain antibodies,^22^ and the structural character of the epitope and interface,^21^ are plausible contributors that our sample sizes do not resolve.

Second, every complex evaluated here is a cognate pair with an experimentally established interaction, so most of our results characterize binding-mode recovery conditional on binding. The interface-mutation and antigen-shuffling analyses partly address this limitation by probing more directly interaction specificity. All models generally reduced confidence for targeted interface mutations and, more strongly, for non-cognate antigen shuffling, but high-confidence decoys remained, particularly among interface mutants. The effect was most pronounced for OpenDDE, which achieved the strongest docking performance yet retained comparatively high confidence for many mutated interfaces. This finding may indicate a greater reliance on learned global sequence–structure patterns than on the precise sequence-specific interactions governing binding energetics. Notably, OpenDDE is the only model evaluated here to incorporate an explicit shape-complementarity loss during training, which rewards favourable cross-chain surface orientation, inter-chain spacing and clash-free contacts. This geometric supervision could therefore contribute to maintaining confidence for interface mutations that preserve the overall complementarity of the predicted complex despite perturbing specific residue-level interactions. Such sensitivity to antigen mutations is relevant when discriminating closely related targets, such as antigenic variants or orthologues from different species, where small sequence changes can substantially alter antibody binding, and for affinity maturation and *de novo* design screening, where candidate ranking may depend on subtle sequence changes at the interface. However, because these decoys are expected, rather than experimentally shown, to bind weakly or not at all, fully closing the gap requires a prospective study of antibody–antigen pairs absent from the training data of the models, with reliable experimental labels of binding and non-binding.

Taken together, our results show that Protenix-v2 offers a useful balance for antibody-design campaigns, combining high docking accuracy with shorter runtimes than OpenDDE and improved rejection of interface mutants. OpenDDE remains preferable when maximizing docking accuracy is the primary objective, but its higher computational cost and greater tendency to assign high confidence to decoys should be considered in large-scale screenings. ESMFold2-fast offers the fastest inference and strong confidence-based discrimination, alongside good decoy rejection. However, it has the lowest docking accuracy and the weakest selection of the best candidate within samples (median NDCG@1, 0.69, Figure 3d). Within its intended scope, the framework we presented provides a current, reproducible reference point for selecting and configuring the latest co-folding models in antibody discovery and engineering, and a basis against which future models can be compared.

## 3. Materials and methods

### 3.1. Construction of the reference and benchmark datasets

#### 3.1.1. Parsing SAbDab2

The complete SAbDab2^25^ summary table and corresponding raw mmCIF structures were downloaded on 20 June 2026 (https://sabdab.opig.stats.ox.ac.uk/). The snapshot contained 21,237 antibody instances from 11,085 PDB entries.

Structures were converted from mmCIF to PDB and standardized by retaining the first model for NMR inputs, removing non-protein atoms, resolving alternative conformations and residue numbering, and excluding failed structures. Missing atoms, if any, were not reconstructed. Fv and V_H_H domains were processed separately and aligned to the 149-position AHo scheme^33^ using the implementation of ANARCI^34^ in AbNatiV2.^35^ CDR1, 2, and 3 were defined as residues at AHo positions 27–42, 57–69 and 108–138 respectively for both V_H_ and V_L_ domains.

Antigens were required to share a biological assembly with the antibody and contact at least three CDR positions within 4.5 Å. Antigen sequences were clustered at 95% identity. Structures with resolution worse than 4 Å or missing density at any core AHo positions 4–145 were excluded, while NMR structures without assigned numerical resolution were retained. For each unique antibody sequence, a representative structure was selected, prioritizing antigen presence (i.e., bound antibody structure rather than unbound), variable-domain completeness (no missing residues), higher resolution, and fewer missing residues in general (including in the antigen), in this order.

To minimize antibody-sequence leakage, each held-out benchmark antibody was required to differ from every reference antibody by at least five substitutions across the combined CDRs and at least three substitutions within H-CDR3. For both comparisons, the normalized BLOSUM62 chemical distance was also required to exceed 50. Entries failing any criterion were moved into the reference set, and distances were recalculated iteratively until convergence. Antigen-sequence leakage was controlled using 95%-identity sequence clusters: a held-out complex was reassigned to the reference set when all its antigen chains belonged to clusters already represented in the reference set. This procedure was likewise repeated until no further reassignments occurred.

Using a deposition-date cutoff of 1 December 2024 and the leakage filtering described above, the resulting split contained 4,489 Fv and 1,009 V_H_H reference structures, together with 91 Fv and 43 V_H_H held-out structures. To limit computational cost, SAbDab2-Bench retained held-out complexes containing exactly one antigen chain shorter than 600 residues, yielding 53 Fv and 35 V_H_H complexes. In the final datasets, the earliest SAbDab2-Bench deposition is 5 December 2024 and the latest Ab-FoldBench deposition is 27 August 2024. Restricting the reference set to structures deposited on or before the common 30 September 2021 model cutoff produced SAbDab2-Ref, comprising 2,611 Fv and 497 V_H_H structures.

#### 3.1.2. Parsing FoldBench

The antibody-antigen subset of FoldBench^20^ comprised 172 chain-level interfaces across 113 PDB entries with upper resolution thresholds of 2.5 Å, where each interface represents one antibody-chain-antigen-chain pair (i.e., a single Fv-antigen complex may contribute two separate heavy-chain and light-chain interfaces). FoldBench PDB identifiers were mapped to the corresponding structures processed through the SAbDab2 pipeline described above. Within each PDB entry, identical antibody-antigen sequence pairs arising from crystallographic copies were collapsed. All antigen chains were preserved, and no antigen-length cutoff was applied. This reconstruction yielded 119 expected antibody-antigen complexes. Six entries (8DTN, 8F5N, 8FXV, 8R4U, 8VZO and 8ZP2) failed the core-density criterion, while 8XNH was absent from the original SAbDab2 snapshot used in this study. The resulting Ab-FoldBench set therefore contained 112 complexes (68 Fvs and 44 V_H_Hs) from 106 PDB entries.

#### 3.1.3. Mutating SAbDab2-bench

Epitope mutants were generated for each complex in SAbDab2-Bench using the procedure detailed below. A schematic of the procedure is shown in Figure 4a.

Epitope residues were defined as antigen residues with a fully resolved backbone and at least one atom within 4.5 Å of an antibody atom. They were then ranked by the extent to which their side chain contacted the antibody and the extent to which they were buried upon binding. This ranking was performed to identify residues whose mutation is more likely to be disruptive to binding.

To do this, a contact score (C) and a burial score (B) were calculated for each epitope residue and their geometric mean served as the interaction ranking score (IR).

(2) $$C_{i}\;=\;\frac{n_{i}}{n_{j}}$$

$n_{i}$ is the number of distinct antibody residues with at least one atom within 4.5 Å of a side-chain atom of epitope residue i. Residue j is the epitope residue making the most such contacts ($n_{j}$) in that complex. Contacts are counted from side-chain atoms only with the exception of glycine whose Cα atom was used instead.

(3) $$B_{i}\;=\;\frac{{S\mathrm{ASA}}_{i}^{u\mathrm{nbound}}{-S\mathrm{ASA}}_{i}^{b\mathrm{ound}}}{{S\mathrm{ASA}}_{i}^{u\mathrm{nbound}}}$$

${SASA}_{i}^{bo\mathrm{und}}$ and ${SASA}_{i}^{un\mathrm{bound}}$ are the solvent-accessible surface areas of residue i in complex and with the antibody removed, computed using the Shrake–Rupley algorithm as implemented in Biopython (Bio.PDB.SASA.ShrakeRupley).^36,37^ If ${SASA}_{i}^{un\mathrm{bound}}$ was 0 meaning that residue i remained completely buried when the antibody was removed, then $B_{i}$ was set to 0.

(4) $$IR_{i}\;=\;\sqrt{C_{i}\;B_{i}}$$

$IR_{i}$ is the geometric mean of the two scores and ensures that highly ranking residues need to make a large number of antibody contacts *and* be substantially buried by the antibody. The interaction ranking score distribution (IR) for all epitope residues in SAbDab2-Bench including the chosen mutation sites is shown in **Figure S10**. If N epitope mutations were being made then the top N residues based on their IR were used as mutation sites.

Once mutation sites were chosen, the BLOSUM62 matrix was used to sample dissimilar amino acids. Each site was substituted with an amino acid drawn from those scoring ≤−1 against the wild type amino acid. Three conditions were run, mutating the top 2, 3 and 5 ranked residues, with three variants generated per condition.

A similar process was applied to identify paratope residues and generate paratope mutants, with the contact and burial scores being calculated with respect to the antigen.

#### 3.1.4. Shuffling SAbDab2-bench

The shuffling procedure was carried out separately for the Fvs and V_H_Hs in SAbDab2-Bench. All non-cognate antigens that bound to the same antibody type (Fv or V_H_H) were considered for shuffling with a given antibody. An epitope motif search and CDR diversity check (schematic in Figure 4e) were performed to mitigate the chance of the shuffled complex being a false negative (a binding pair).

The epitope motif served to identify non-cognate antigens that were unlikely to have an epitope similar to the cognate epitope. This was performed using Folddisco, a structural motif search tool.^38^ Following the methodology detailed in the Folddisco paper,^38^ a custom index was built from all antigens, and an antibody’s cognate epitope served as the query motif. Searches were run in pre-filter-only mode (–skip-match) with default settings. Additionally, to make the search more lenient, alternate amino acid identities for the query epitope residues were permitted. These alternate amino acid identities were restricted to those that scored greater than 0 in the BLOSUM62 substitution matrix. This search provided an idf score for all antigens (cognate and non-cognate) in the index with respect to the query cognate epitope. The idf score is a measure of the significance in overlapping structural features between the query structural motif and a given structure. The idf score and the structural features used by Folddisco are explained further in the Folddisco paper.^38^ Folddisco does not report idf scores for structures with no features in common with the query. Such cases were treated as having an idf score of 0. Non-cognate antigens with an idf score in the bottom quartile were deemed not to have significant overlapping structural features with the cognate epitope and passed this check.

The CDR diversity check made sure that antibodies that bind to the non-cognate antigens had diverse CDR regions when compared to the antibody the non-cognate antigens were being shuffled with. This was done by ensuring that the antibodies that bind to the non-cognate antigens differed from the antibody to be shuffled with by at least five substitutions across the combined CDRs and at least three substitutions within H-CDR3. Additionally, the normalized BLOSUM62 chemical distance across the H-CDR3 and the combined CDRs had to both exceed 50.

For each antibody from the dataset 5 non-cognate antigens were chosen from those that passed both checks. An effort was made to sample the 5 non-cognate antigens from different length bins (<20, 20–100, 100–300, 300–500 and >500 residues) with the non-cognate antigen with the lowest idf score in each bin getting priority. It was also made sure that 5 non-cognate antigens were members of different clusters when clustering all SAbDab2 antigens at 80% sequence identity. The pooled distribution of idf scores corresponding to the searches conducted with all the cognate epitopes from SAbDab2-Bench is shown in Figure S10.

### 3.2. Multiple-sequence alignments

#### 3.2.1. Unpaired MSAs

For each benchmark subset, we precomputed a separate unpaired multiple-sequence alignment (MSA) for each protein chain using the ColabFold MMseqs2 workflow.^39,40^ Searches queried the UniRef30 2023–02 snapshot (uniref30_2302_db) and the ColabFold environmental sequence database 2021–08 snapshot (colabfold_envdb_202108_db). The MMseqs2 searches were GPU-accelerated and used eight CPU threads, three iterative search rounds, an E-value cutoff of 0.1, and at most 10,000 prefilter candidates per query (–max-seqs 10,000). Hits to database cluster representatives were expanded to their member sequences and realigned.

For alignments containing at least 1,000 sequences, MSA diversity filtering was enabled using – filter-msa 1 and – filter-min-enable 1000. Filtering used – diff 3000, –qid thresholds of 0.0, 0.2, 0.4, 0.6, 0.8, and 1.0, –qsc 0, and – max-seq-id 0.95. The filtered UniRef30 and environmental alignments were then combined, with the query sequence retained as the first entry. These chain-wise unpaired MSAs were used by all MSA-enabled models, including ESMFold2.

#### 3.2.2. Paired MSAs

For models supporting paired-MSA inputs, paired alignments were also generated using the ColabFold MMseqs2 workflow. In the role-scoped condition, sequences were paired separately among chains belonging to the same interaction partner: binder chains were paired with one another, and target chains were paired with one another. Thus, binder and target sequences were not paired across the interface. In the complex-scoped condition, all protein chains were paired together, including across the binder-target interface.

The paired and unpaired alignments were supplied together to AlphaFold 3. Protenix and OpenDDE used the role-scoped paired alignments in their respective MSA-enabled conditions. ESMFold2 used only the unpaired alignments because it does not provide a paired-MSA input. The same precomputed MSA artifacts were reused across the corresponding model runs.

### 3.3. Template search

For the template-enabled models (AlphaFold 3, Protenix, and OpenDDE), we implemented a unified automatic template-search workflow so that each model received the same selected templates and query-template alignments in a backend-compatible form. ESMFold2 was not supplied with templates. The search strategy was adapted from Protenix,^17^ which itself follows the AlphaFold 3 template-search procedure.^16^

For a given protein chain (query), we first built a profile hidden Markov model (HMM) using hmmbuild from the query’s paired and unpaired MSAs. If both are available, they are concatenated, keeping the paired MSA first; otherwise only the unpaired MSA is used. The MSA input is always cropped to the first 300 sequences. The query sequence alone is used as input if the MSA has fewer than 10 sequences. The profile HMM is searched with hmmsearch (flags –noali –F1 0.1 –F2 0.1 –F3 0.1 -E 100 –incE 100 –domE 100 –incdomE 100) against a FASTA file of protein sequences from a 2022–09-28 PDB snapshot,^41,42^ restricted to entries with corresponding structures in a local PDB mmCIF mirror (dated 2024–05-22). The snapshot and mirror dates determine only which entries are retrievable; eligibility as a template was governed by release-date and other filters explained below.

Hits were processed in E-value-ranked order. A hit required a PDB release date on or before 2021–09-30, which coincides with the common model-training cutoff and therefore excludes every Ab-FoldBench and SAbDab2-Bench structure, together with an aligned length of at least 10 residues and more than 10% query coverage, to be considered a candidate. Hits that were exact subsequences of the query covering more than 95% of its length were excluded and the resulting candidate hits were deduplicated. The 20 highest-ranked candidate hits were carried forward for structural validation.

Each candidate hit’s sequence (extracted from its corresponding local mmCIF file) was realigned to the query sequence using Kalign v3.6.0. Candidates for which mmCIF parsing or realignment failed were discarded; the rest underwent structural validation, which required them to meet the following criteria: no distances greater than 150 Å between consecutive resolved Cα atoms in the aligned template chain and at least five resolved template atoms mapped to query residues. This validation was performed in rank order until four valid templates were retained or all 20 candidates were processed.

### 3.4. Model implementation and compute specifications

The five structure-prediction models that were evaluated in this study were installed in dedicated conda environments, with the exception of AlphaFold 3, which required building a Docker container. ESMFold2 was run entirely unmodified via its repository, with no source-code changes. AlphaFold 3, Protenix-v2, and OpenDDE were each cloned from their repositories and patched before installation. The repository and commit hash at which each model was cloned from are shown in Table S4. For Protenix-v2, we used the repository state associated with its 8 April 2026 release (commit 2475421477ab414b571149ad4a875c390ff8a35d; https://github.com/bytedance/Protenix/). The Protenix-v2 model weights were downloaded at that time using the checkpoint URL specified in this repository state (https://protenix.tos-cn-beijing.volces.com/checkpoint/protenix-v2.pt), which carried the statement “The Protenix project including both code and model parameters is released under the Apache 2.0 License”. Patches made to AlphaFold 3, Protenix-v2, and OpenDDE source code served two main purposes: (i) to enable the utilisation of the unified template search described in Section 3.3, and (ii) to perform metric calculations in accordance with Section 3.6. Additional patches were also made to standardize input and output formats across all models for user convenience. The results presented in this study should be representative of how these models perform as no patches altered code for inference.

Inference for all folding models was performed on the Isambard-AI Phase 2 facility, which provides NVIDIA GH200 120GB GPUs running CUDA Version 12.7 paired with ARM (aarch64) CPUs.

#### 3.4.1. Compatible installation

Since the folding models were likely developed on the more prevalent x86_64 CPU architecture, certain dependencies required modified version pins before installation would succeed for our compute specifications. These changes are listed in Table S5.

#### 3.4.2. AlphaFold 3 container

An AlphaFold 3 Docker container (https://hub.docker.com/r/arnavcheruku/foldingsuite-af3) was built on a machine with an aarch64 CPU, following the installation instructions provided in the AlphaFold 3 repository. The Docker container was converted to a Singularity container prior to inference as the Isambard-AI facility does not support Docker.

#### 3.4.3. Missing ESMFold2 dependencies

ESMFold2, which was previously reported as being faster than AF3,^18^ was slower than its competitor in this study. This may partly reflect the absence of optional acceleration libraries such as flash-attn, xformers, and transformer-engine, which caused ESMFold2 to use PyTorch fallback implementations.

### 3.5. Model inference settings

The five co-folding models were evaluated using the configurations summarized in Table 1. Additional model-specific settings, including automatic versus manually specified templates and alternative MSA pairing strategies, were evaluated separately (Figure S2). Only the configurations in Table 1 were used for the primary analyses. Unless otherwise stated, each model generated five samples for each of ten seeds, yielding 50 predictions per target. The numbers of recycling and sampling steps followed the corresponding model recommendations.

### 3.6. Structure-quality and confidence metrics

To ensure consistent comparisons across co-folding models, all predictions were evaluated using the same analysis workflow. Throughout, let B denote the residues in all binder chains (i.e., the V_H_ and V_L_ domains in Fvs or the V_H_H domain in nanobodies), and let T denote the residues in all target chains (i.e., the antigen chains). The total number of residues is $N=\left|B\right|+\left|T\right|.$

#### 3.6.1. DockQ

DockQ was calculated using DockQ v2.1.3^26,27^:

(5) $$\mathrm{Dock}Q^{B↔T}=\frac{1}{3}\left[f_{\mathrm{nat}}+\frac{1}{1+{\left(\mathrm{iRMSD}/1.5\right)}^{2}}+\frac{1}{1+{\left(\mathrm{LRMSD}/8.5\right)}^{2}}\right].$$

To account for multi-chain binders and targets, DockQ was calculated for the combined interface between all binder chains and all target chains. Here, $f_{\mathrm{nat}}$ was calculated from all native contacts between B and T; iRMSD was the backbone root mean square deviation (RMSD) over all native interface residues in B∪T; and LRMSD was the backbone binder RMSD of B after least-squares superposition of the predicted target group onto the native target group. For targets containing sequence-identical, symmetry-equivalent antigen chains, such as homodimers, all valid permutations of the equivalent chains were evaluated, and the highest DockQ was reported.

#### 3.6.2. Interface confidence

We calculated the interface predicted template modeling (ipTM),^2,16^ an AlphaFold-Multimer^2^ confidence metric derived from each model’s output predicted aligned error (PAE) matrix. The binder-to-target component was calculated as:

(6) $$\mathrm{ipT}M^{B\to T}=\underset{i\in B}{\max}\frac{1}{\left|T\right|}\underset{j\in T}{\sum }\overset{N_{\mathrm{bins}}}{\underset{b=1}{\sum }}p_{ij}^{b}{\left[1+{\left(\frac{\Delta_{b}}{d_{0}\left(N\right)}\right)}^{2}\right]}^{-1},$$

where $p_{ij}^{b}$ is the predicted probability of the PAE bin b for residue j aligned on residue i, $\Delta_{b}$ is the corresponding bin centre among $N_{\mathrm{bins}}$ bins, and $d_{0}\left(N\right)=1.24{\left[\max\left(N,19\right)-15\right]}^{1/3}-1.8.$ The target-to-binder score, $\mathrm{ipT}M^{T\to B},$ was calculated by exchanging B and T. We defined:

(7) $$\begin{array}{cc} {\mathrm{ipTM}}_{min}^{B↔T} & =\min\left(\mathrm{ipT}M^{B\to T},\mathrm{ipT}M^{T\to B}\right),\\ {\mathrm{ipTM}}_{max}^{B↔T} & =\max\left(\mathrm{ipT}M^{B\to T},\mathrm{ipT}M^{T\to B}\right). \end{array}$$

We additionally calculated the interaction prediction score from aligned errors (ipSAE) from the output PAE matrix following Dunbrack *et al*.^43^ as implemented in.^44^ The key difference is that ipTM converts each possible $N_{\mathrm{bins}}$ aligned error to a score before averaging over its predicted distribution, whereas ipSAE first calculates

(8) $$\mathrm{PA}E_{\mathrm{ij}}=\overset{N_{\mathrm{bins}}}{\underset{b=1}{\sum }}p_{ij}^{b}\Delta_{b}$$

and then converts this expected error to a score. Thus, the pairwise contributions are

(9) $$\mathrm{ipTM}:\;\overset{N_{\mathrm{bins}}}{\underset{b=1}{\sum }}p_{ij}^{b}{\left[1+{\left(\frac{\Delta_{b}}{d_{0}\left(N\right)}\right)}^{2}\right]}^{-1}\;\;\mathrm{and}\;\;\;\mathrm{ipSAE}:\;{\left[1+{\left(\frac{\mathrm{PA}E_{\mathrm{ij}}}{d_{0,i}}\right)}^{2}\right]}^{-1}.$$

ipSAE additionally uses only residue pairs with PAE<10 Å and calculates $d_{0,i}$ from the number of such pairs rather than from the total complex size. We defined:

(10) $$\begin{array}{cc} {ip\mathrm{SAE}}_{min}^{B↔T} & =\min\left(\mathrm{ipSA}E_{B\to T},\mathrm{ipSA}E_{T\to B}\right),\\ {ip\mathrm{SAE}}_{max}^{B↔T} & =\max\left(\mathrm{ipSA}E_{B\to T},\mathrm{ipSA}E_{T\to B}\right). \end{array}$$

#### 3.6.3. Confidence-based selection and best observed performance

For each target complex, predictions from all seeds and diffusion samples were ranked using the same ranking score

(11) $$R\;=\;0.8\;{\mathrm{ipTM}}_{max}^{B↔T}+0.2\;\mathrm{pTM},$$

where ${\mathrm{ipTM}}_{max}^{B↔T}$ is defined above and pTM is the global counterpart of ipTM which includes both intra-chain and inter-chain residue pairs across the complete complex. Exact ties were resolved by selecting the lowest seed and then the lowest diffusion sample index. Model-specific ranking scores were retained for reporting but were not used for this common selection procedure. pTM has the same definition across all models because it does not require binder-target grouping. The pTM value was therefore takendirectly from each model’s output.

We denote by ${Do\mathrm{ckQ}}_{se\mathrm{lected}}^{B↔T}$ the DockQ score of the prediction selected using the ranking R. We denote by ${Do\mathrm{ckQ}}_{\mathrm{best}}^{B↔T}$ the highest DockQ observed across all seeds and diffusion samples. ${Do\mathrm{ckQ}}_{se\mathrm{lected}}^{B↔T}$ therefore measures confidence-based selection without access to the native structure, whereas ${Do\mathrm{ckQ}}_{\mathrm{best}}^{B↔T}$ represents the best observed performance within the generated ensemble.

### 3.7. Benchmark evaluation and uncertainty estimation

#### 3.7.1. Seed-subset averaging and complex-level uncertainty

All 252 subsets of five seeds were evaluated, with five samples per seed. For each complex and model, success indicators were averaged across seed subsets. Reported pass rates are the means of these per-complex success fractions. We estimated 95% percentile confidence intervals using 10,000 bootstrap resamples of complexes with replacement. Thus, the intervals quantify variation across complexes after averaging over seed subsets.

#### 3.7.2. Pairwise comparison between models

All five models were evaluated on the same 200 complexes, so comparisons between them are paired and were analyzed as such. For each model and target, the single prediction selected by the common ranking score R across all ten seeds and five diffusion samples was used, so that each model contributes exactly one ${Do\mathrm{ckQ}}_{se\mathrm{lected}}^{B↔T}$ value per target. Note that the resulting pass rates correspond to the full ten-seed budget and therefore differ slightly from the five-of-ten seed subset means discussed in the previous section and reported in Figure 2c.

Differences in pass rate were assessed with McNemar’s exact test. For a pair of models, targets on which both succeed or both fail carry no information about their relative performance, so the test considers only the discordant targets: letting $n_{\mathrm{AB}}$ and $n_{\mathrm{BA}}$ denote the numbers of targets passed (${Do\mathrm{ckQ}}_{se\mathrm{lected}}^{B↔T}>0.23$) by one model but not the other, the two-sided P value is that of a binomial test of $n_{\mathrm{AB}}$ successes out of $n_{\mathrm{AB}}+n_{\mathrm{BA}}$ trials against a null probability of one half (equal performance, i.e. $n_{\mathrm{AB}}=n_{\mathrm{BA}}$).

Differences in median ${Do\mathrm{ckQ}}_{se\mathrm{lected}}^{B↔T},$ the statistic shown in Figure 2b, were assessed by a paired permutation test. Under the null hypothesis that two models are exchangeable within a target, the pair of values recorded for each complex may be swapped at random; 20,000 such permutations were drawn, the difference in medians recomputed for each, and the two-sided P value taken as $\left(1+\#\{\left|\delta^{*}\right|\geq \left|\delta \right|\}\right)/\left(1+20,000\right),$ where δ is the observed difference. The same resampled indices were applied to both models so that the pairing is preserved.

Both tests were applied to all $\left(\begin{array}{c} 5\\ 2 \end{array}\right)=10$ model pairs, and the resulting *p*-values were adjusted separately for each test using the Benjamini–Hochberg procedure to control the false discovery rate across the ten comparisons.

#### 3.7.3. Inference-time scaling

Inference-time scaling was evaluated for seed budgets from one to ten. For each budget b, all $\left(\begin{array}{c} 10\\ b \end{array}\right)$ seed subsets were evaluated using five diffusion samples per seed. Candidates were ranked by R, and the top candidate was considered successful when DockQ>0.23. For each target, the pass probability was calculated across all subsets. Lines show the mean pass probability across targets, and shaded regions show 95% percentile confidence intervals from 10,000 bootstrap resamples of complexes, after averaging success across all seed subsets at each budget.

#### 3.7.4. Confidence-ranking performance

For each model and target, all 50 predictions were ranked by R. Ranking performance was measured using normalised discounted cumulative gain at rank one (NDCG@1):

(12) $$\mathrm{NDCG}@1=\frac{{D\mathrm{ockQ}}_{s\mathrm{elected}}^{B↔T}}{{D\mathrm{ockQ}}_{b\mathrm{est}}^{B↔T}},$$

where ${Do\mathrm{ckQ}}_{se\mathrm{lected}}^{B↔T}$ is the DockQ of the model-selected prediction and ${Do\mathrm{ckQ}}_{\mathrm{best}}^{B↔T}$ is the highest DockQ among the same 50 predictions. NDCG@1 therefore measures how effectively the confidence score selects the best available structure and ranges from zero to one, with one indicating perfect selection. It was treated as undefined when ${Do\mathrm{ckQ}}_{\mathrm{best}}^{B↔T}=0.$

### 3.8. Non-cognate antigen-template retrieval and selection

For each antigen, we searched the experimental PDB and retained templates with an exact sequence match across the antigen region. Additional terminal residues were permitted. We excluded the native entry and structures containing the cognate antibody or a similar antibody, defined by at least 95% heavy-chain sequence identity. Candidates were required to contain all antigen components in one biological assembly, with at least 95% backbone coverage and no missing backbone atoms in the cognate epitope. No resolution cutoff was applied.

Apo structures, including those containing only buffer additives or physiological cofactors such as native metal ions or hemes, were prioritized over structures bound to other proteins, nucleic acids or potentially conformation-changing ligands, such as substrates or inhibitors. Candidates were then ranked by backbone coverage, coordinate coverage, resolution and release date. One template was selected per complex, yielding 51 apo and 68 potentially conformation-changing templates. RMSD to the native antigen was not used for selection but is reported in Figure S3a. The list of selected templates is available in the publication’s GitLab repository (https://gitlab.doc.ic.ac.uk/sormanni-lab/cofolding-benchmarking).

### 3.9. Inter-run variability

To test whether Protenix-v2 runs are deterministic when the seeds and sampling settings are fixed, we folded 5 nanobody-antigen complexes across 3 identical runs with hard-coded seeds. This analysis revealed complex-dependent variation in the output across the runs. Further investigation revealed that achieving determinism required an additional deterministic argument in the seed settings to be set to true, since several PyTorch algorithms are non-deterministic by default and therefore introduce non-determinism even when the seed and sample settings are fixed. The maximum absolute difference in ipTM and pTM metrics across the 3 runs for each complex, under both the deterministic and non-deterministic settings, is shown in Table S6. As expected, the deterministic setting eliminates run-to-run variation entirely, with the maximum difference in metrics always equal to 0. However, this determinism comes at the cost of speed, with deterministic runs taking approximately 1.2× the runtime of non-deterministic runs. This default non-deterministic behavior may be a general feature of these co-folding models and could offer a potential explanation for why benchmarking of these models can be difficult to reproduce. Therefore, the determinism of the different models and the complex-dependent behavior of the observed non-determinism in Protenix-v2 should be investigated in the future.

## Supplementary Material

supplementary_information.docx

## Data Availability

All Python scripts used to perform the analyses in this study, together with the PDB structures from Ab-FoldBench and SAbDab2-Bench, their non-cognate antigen templates and the corresponding prediction metrics for each seed, sample and model, are available at https://gitlab.doc.ic.ac.uk/sormanni-lab/cofolding-benchmarking. All the generated structures in their .cif format are available on Zenodo at https://zenodo.org/records/22874923.
