# Supplementary material for: Beyond AlphaFold 3: a standardised benchmark of open-source co-folding models for antibody–antigen complexes, from docking accuracy to interaction specificity
Source: MAbs. 2026 Nov 12;18(1):2760737. doi: 10.1080/19420862.2026.2760737 (PMC13761387; doi:10.1080/19420862.2026.2760737)
Supplement: Supplementary file 1 [file KMAB_A_2760737_SM5070.docx]

Supplementary Information

Beyond AlphaFold 3: a standardised benchmark of open-source co-folding models for antibody-antigen complexes

Aubin Ramon^1^, Arnav Cheruku^1^, Haowen Zhao^1,2^, Pietro Sormanni^1,†^

^1^ Department of Chemical Engineering, Imperial College London, London, UK

^2^ Yusuf Hamied Department of Chemistry, University of Cambridge, Cambridge, UK

† Corresponding author: pietro.sormanni@imperial.ac.uk

**Supplementary Figure S1. Additional benchmark database statistics.** (**a**-**b**) Bar plots of count (**a**) and percent composition (**b**) of Fv (i.e., V_H_-V_L_ antibodies) and V_H_H (i.e., single-domain antibodies) in each dataset considered. Pooled refers to SAbDab2-Bench and Ab-FoldBench combined. (**c**) Swarmplots of the H-CDR3 length distributions in the three datasets (see legend) for Fv and V_H_H separately (x-axis). (**d**) like **c** but reporting antigen length. Black lines in the box plots indicate medians, and black stars indicate means. Points represent individual complexes and are randomly subsampled where necessary for visualisation.

**Supplementary Figure S2. Effects of MSA and template settings on antibody–antigen co-folding performance.** Models were evaluated on 200 complexes pooled from SAbDab2-Bench and Ab-FoldBench, without MSAs, with MSA pairing restricted to chains within the antibody (Ab) and antigen (Ag), or with MSAs paired across the complete complex (legend); ESMFold2 used its supported unpaired-MSA mode. Hatching indicates whether templates were omitted, retrieved automatically for both Ab and Ag, or retrieved automatically for the Ab while the known Ag structure was supplied manually. In the latter setting, only antigen coordinates were provided, without the antibody or its binding pose. Bars show $\text{DockQ}_{\text{selected}}^{B\text{↔}T}$ pass rates, partitioned into acceptable ($0.23\text{<}x\text{≤}0.49$), medium ($0.49\text{<}x\text{≤}0.80$) and high-quality ($0.80\text{<}x\text{≤}1.00$) predictions, where $x\text{=}\text{DockQ}_{\text{selected}}^{B\text{↔}T}\text{,}$ across all five-of-ten seed subsets, with five samples per seed; error bars show 95% bootstrap confidence intervals over complexes (10,000 resamples), using seed-subset-averaged success rates.

**Supplementary Figure S3. Effects of non-cognate antigen templates on antibody–antigen co-folding performance.** Analyses used 119 complexes pooled from SAbDab2-Bench and Ab-FoldBench for which an alternative antigen structure, determined without the antibody being evaluated, was identified in the PDB (i.e., apo or bound to another protein or substrate; see **Methods**). (**a**) C$\alpha$ RMSD between the non-cognate template and the cognate antibody-bound antigen, calculated after independent superposition of either all antigen residues or only epitope residues. Epitope residues were defined by a heavy-atom distance of $\text{≤}4.5$ Å from the antibody in the native complex. Points represent complexes, with lines connecting paired measurements. Black lines in the box plots indicate medians, and black stars indicate means. (**b**) Co-folding performance of OpenDDE, Protenix-v2 and AF3 on the same complexes using automatic templates, the manually supplied cognate-bound antigen (cross-hatching), or the non-cognate antigen template (hatching). In both manual-template settings, antibody templates were retrieved automatically. Bars show $\text{DockQ}_{\text{selected}}^{B\text{↔}T}$ pass rates, partitioned into acceptable ($0.23\text{<}x\text{≤}0.49$), medium ($0.49\text{<}x\text{≤}0.80$) and high-quality ($0.80\text{<}x\text{≤}1.00$) predictions, where $x\text{=}\text{DockQ}_{\text{selected}}^{B\text{↔}T}\text{.}$ Bars show mean pass rates across all five-of-ten seed subsets, with five samples per seed; error bars show 95% bootstrap confidence intervals over complexes (10,000 resamples), using seed-subset-averaged success rates.

**Supplementary Figure S4. Comparison of OpenDDE model weights.** The standard general-purpose (general) and antibody-antigen-specific (abag) OpenDDE weights were compared in a separate control run on the 200 complexes pooled from SAbDab2-Bench and Ab-FoldBench. This run used unpaired MSAs and automatic template retrieval for both the antibody and antigen (which cannot retrieve identical sequences, see Methods). We used here five seeds with five samples per seed, yielding 25 candidates per target. The prediction with the highest ranking score was selected for each target. (left panel) Overall $\text{DockQ}_{\text{selected}}^{B\text{↔}T}$ pass rates, partitioned into acceptable, medium- and high-quality predictions. (right panel) Pass rates separated by antibody format. Bold labels show total pass rates, and labels within the bars give the number of complexes in each DockQ category.

**Supplementary Figure S5. Benchmark performance on the individual test sets.** Results are shown separately for (**a**) SAbDab2-Bench (88 complexes) and (**b**) Ab-FoldBench (112 complexes). The analyses, metric definitions and graphical conventions follow **Figure 2** in the main manuscript.

**Supplementary Figure S6. Comparison of co-folding runtimes on H100 and RTX PRO 6000 GPUs.** Distributions of runtime for 63 matched SAbDab2-Bench complexes (39 Fv and 24 V_H_H) on (**a**) H100 and (**b**) RTX PRO 6000 GPUs. Folding times correspond to 5 seeds with 5 samples per seed per complex and include MSA and template searches, where applicable. Points represent individual complexes. Black lines indicate medians, stars indicate means. Values above each distribution indicate the total runtime across all 63 complexes. ESMFold2 runtimes may have been affected by the absence of optional acceleration libraries flash-attn, xformers, and transformer-engine in both configurations.

**Supplementary Figure S7. Associations between antigen length, experimental method, resolution and co-folding performance.** Analyses used 200 complexes pooled from SAbDab2-Bench and Ab-FoldBench, with Fv and V_H_H combined. (**a**-**b**) $\text{DockQ}_{\text{best}}^{B\text{↔}T}$ versus total antigen length (**a**) and reported experimental resolution (**b**). For each model and target, the highest DockQ among the 50 predictions was used. Colored points show means across five equal-count bins of 40 complexes, with horizontal and vertical error bars indicating SEM. Dashed lines show linear fits through the bin means, using log-transformed antigen length in **a**. Grey points represent individual target/model values. Annotations report the mean $\text{±}$ standard deviation of the five model-specific Spearman correlations, each calculated over all 200 complexes rather than the bin means. (**c**) Distributions of antigen length (left) and $\text{DockQ}_{\text{best}}^{B\text{↔}T}$ averaged across the five models for each complex (right), grouped by X-ray crystallography (156 complexes) or cryo-EM (44 complexes). Points represent individual complexes. Black lines in the box plots indicate medians. (**d**) Antigen length versus reported resolution, with points coloured by experimental method (legend). The dashed line shows a linear fit of log-transformed antigen length against resolution across all 200 complexes; the annotation reports the corresponding Spearman correlation. Antigen length is shown on a logarithmic axis in **a**, **c** (left) and **d**. Antigen length overlaps with experimental method, resolution and antibody format; consequently, the observed association does not establish an independent effect of antigen size.

**Supplementary Figure S8. Comparison of prediction-time confidence metrics.** Models were evaluated on 200 complexes pooled from SAbDab2-Bench and Ab-FoldBench as in the primary analysis. For each model and target, the prediction with the highest common ranking score, $R\text{=}0.8 \text{ipTM}_{\text{max}}^{B\text{↔}T}\text{+}0.2 \text{pTM}\text{,}$ was selected from the 50 candidates generated across ten seeds and five diffusion samples. Bars show the Spearman correlation between $\text{DockQ}_{\text{selected}}^{B\text{↔}T}$ and five prediction-time confidence metrics across the 200 complexes. Minimum and maximum ipTM and ipSAE refer to the lower and higher values, respectively, across the two binder-target directions; H-CDR3 pLDDT is the mean predicted local-distance difference test score over the heavy-chain CDR3 residues in the predicted complex as output by each model. Bars are grouped and coloured by model, while hatching identifies the confidence metric. Higher Spearman correlation indicates stronger agreement between the confidence metric and the observed DockQ ranking across targets.

**Supplementary Figure S9. Receiver operating characteristic analysis of confidence-based prediction quality discrimination.** (**a**) Receiver operating characteristic (ROC) curves distinguishing predictions with $\text{DockQ}_{\text{selected}}^{B\text{↔}T}\text{≤}0.49$ from those with $\text{DockQ}_{\text{selected}}^{B\text{↔}T}\text{>}0.80\text{.}$ (**b**) ROC curves distinguishing predictions with $\text{DockQ}_{\text{selected}}^{B\text{↔}T}\text{≤}0.23$ from those with $\text{DockQ}_{\text{selected}}^{B\text{↔}T}\text{>}0.49\text{.}$ Both comparisons use $\text{ipTM}_{\text{max}}^{B\text{↔}T}\text{.}$ Predictions between the stated thresholds were excluded from each comparison. Legends report the area under the ROC curve and dashed diagonals indicate the random baseline (ROC-AUC = 0.50). Corresponding PR curves are shown in **Figure 3b** in the main manuscript.

**Supplementary Figure S10. Distribution of interaction ranking scores across epitope residues in SAbDab2-Bench.** Of the 1658 epitope residues identified in SAbDab2-Bench, 1451 had non-zero interaction ranking ($IR$) scores. The remaining 207 made no side-chain-mediated contact with the antibody (C$\alpha$-mediated contact for glycine) or had $\text{SASA}_{i}^{\text{unbound}}\text{=}0\text{.}$ (**a**) Histogram of $IR$ scores across epitope residues. Orange bars show the subset selected as mutation sites under the five-mutation condition. (**b**-**d**) Swarm plots of $IR$ scores, with selected mutation sites highlighted for the $5$- (**b**), $3$- (**c**) and $2$-mutation (**d**) conditions. Points represent individual epitope residues. Residues with $IR\text{=}0$ are excluded throughout.

**Supplementary Figure S11. Pooled distribution of Folddisco** $idf$ **scores across all cognate epitope motif searches.** Fv and V_H_H complexes in SAbDab2-Bench were shuffled separately, so that an Fv epitope was matched only against antigens bound by other Fvs, and likewise for V_H_Hs. Two indexes were therefore built, one per antibody format, and each cognate epitope was searched against the index corresponding to its binder. Within each subset, every query (cognate epitope) and structure (indexed antigen, including the antigen of origin) pair received an $idf$ score; all such scores are plotted by subset. (**a**, **c**) Histograms of $idf$ scores for the Fv and V_H_H subsets. Grey bars show all query-structure pairs, with the non-cognate antigens selected for shuffling in orange and the self-scores, that is, the score of each cognate epitope against its antigen of origin, in blue. (**b**, **d**) Swarm plots of the same distributions for the Fv and V_H_H subsets. Points represent $idf$ scores for individual query-structure pairs, coloured as in **a** and **c**. In both subsets the antigens selected for shuffling score well below the self-scores, indicating limited structural overlap with the cognate epitope compared to the cognate antigen.

**Supplementary Figure S12. Sensitivity to mutation by mutation count.** Analyses and graphical conventions follow **Figure 4**, with 2, 3 and 5 mutations shown separately. (**a**) Distributions of $\Delta$ipTM (WT - mutant). (**b**) Fractions below the high-accuracy cutoffs from **Table 3**. Light bars use all 88 complexes; dark bars use model-specific WT-above-cutoff cohorts. Error bars show 95% bootstrap confidence intervals. Brackets mark BH-adjusted $p\text{<}0.05$ for comparisons calculated separately by mutation count and mutated partner; Ag denotes antigen-only significance.

**Supplementary Figure S13. Sensitivity to mutation and antigen shuffling using docking ipTM cutoffs.** Analyses and graphical conventions follow **Figure 4**, but using the docking (epitope mapping) $\text{ipTM}_{\text{max}}^{B\text{↔}T}$ cutoffs from **Table 3** instead of the high-accuracy cutoffs. Dark bars use the 21 complexes whose WT exceeds the docking cutoff in all five models. Ab and Ag denote antibody- and antigen-only significance, respectively.

**Supplementary Table S1. Model-specific optimal** $\text{ipTM}_{\text{max}}^{B\text{↔}T}$ **cutoffs stratified by antibody format.** AlphaFold 3, Protenix-v2 and OpenDDE use automatic antibody and antigen templates; ESMFold2 variants use no templates. Cutoffs were derived as in Table 3, but separately for the 121 Fv and 79 V_H_H complexes. For each target, the prediction with the highest ranking score, $R\text{,}$ was selected from the 50 candidates generated across all seeds and diffusion samples, and cutoffs maximise Youden’s $J$ for each classification task. Predictions falling between the thresholds of each task were excluded. PR-AUC denotes average precision for $\text{ipTM}_{\text{max}}^{B\text{↔}T}$ as a classifier for each task, computed as in Figure 3b. Values show estimates $\text{±}$ approximate 95% CI half-widths, calculated from 10,000 resamples of complexes. Each resample was drawn from the pooled cohort, with format-specific statistics calculated from its Fv or V_H_H complexes. Because stratification reduces the number of complexes entering each comparison, these cutoffs are intended to assess whether calibration transfers between antibody formats rather than to define format-specific operating points. Under *pooled cutoff*, the threshold derived from all 200 complexes in **Table 3** is applied to both formats. For its bootstrap intervals, the pooled threshold was re-estimated in each resample and applied to both formats within that resample. Calculations used exact cutoffs before rounding for display.

|  |  | **AlphaFold 3** | **Protenix-v2** | **OpenDDE** | **ESMFold2** | **ESMFold2 fast** |
| --- | --- | --- | --- | --- | --- | --- |
| **High-accuracy structure prediction:** $\text{DockQ}_{\text{selected}}^{B\text{↔}T}\text{≤}0.49$ **vs.** $\text{DockQ}_{\text{selected}}^{B\text{↔}T}\text{>}0.80$ | | | | | | |
| **Format-specific cutoff** | | | | | | |
| **PR-AUC** | Fv | $0.99\text{±}0.02$ | $0.88\text{±}0.12$ | $0.88\text{±}0.12$ | $0.93\text{±}0.09$ | $1.00\text{±}0.01$ |
|  | V_H_H | $0.95\text{±}0.06$ | $0.95\text{±}0.06$ | $0.97\text{±}0.04$ | $0.91\text{±}0.11$ | $0.97\text{±}0.05$ |
| **Best** $\text{ipTM}_{\text{max}}^{B\text{↔}T}$ **cutoff** | Fv | $0.85\text{±}0.07$ | $0.88\text{±}0.03$ | $0.91\text{±}0.04$ | $0.79\text{±}0.12$ | $0.86\text{±}0.02$ |
|  | V_H_H | $0.78\text{±}0.09$ | $0.93\text{±}0.06$ | $0.90\text{±}0.04$ | $0.81\text{±}0.05$ | $0.83\text{±}0.09$ |
| **False discovery rate** | Fv | $0.0\text{±}14.5$% | $12.8\text{±}11.2$% | $17.8\text{±}11.8$% | $16.7\text{±}18.3$% | $4.2\text{±}7.9$% |
|  | V_H_H | $9.1\text{±}14.4$% | $11.1\text{±}12.8$% | $14.3\text{±}12.5$% | $16.0\text{±}14.1$% | $4.0\text{±}10.0$% |
| **False negative rate** | Fv | $7.4\text{±}6.0$% | $2.9\text{±}4.4$% | $2.6\text{±}5.1$% | $7.4\text{±}8.1$% | $0.0\text{±}0.7$% |
|  | V_H_H | $9.1\text{±}11.8$% | $4.0\text{±}7.4$% | $0.0\text{±}11.3$% | $4.5\text{±}10.3$% | $4.0\text{±}6.4$% |
| **Pooled cutoff of Table 3 applied to each format** | | | | | | |
| **Pooled** $\text{ipTM}_{\text{max}}^{B\text{↔}T}$ **cutoff** | Fv, V_H_H | $0.78\text{±}0.07$ | $0.91\text{±}0.05$ | $0.90\text{±}0.03$ | $0.79\text{±}0.10$ | $0.83\text{±}0.04$ |
| **False discovery rate** | Fv | $18.2\text{±}17.3$% | $10.8\text{±}11.4$% | $19.6\text{±}11.7$% | $16.7\text{±}15.8$% | $4.2\text{±}9.2$% |
|  | V_H_H | $9.1\text{±}12.3$% | $17.2\text{±}15.3$% | $14.3\text{±}11.6$% | $19.2\text{±}16.3$% | $4.0\text{±}8.0$% |
| **False negative rate** | Fv | $0.0\text{±}5.8$% | $5.7\text{±}6.9$% | $2.6\text{±}4.7$% | $7.4\text{±}10.4$% | $0.0\text{±}0.0$% |
|  | V_H_H | $9.1\text{±}14.1$% | $4.0\text{±}6.9$% | $0.0\text{±}5.1$% | $4.5\text{±}9.8$% | $4.0\text{±}7.7$% |
| **Docking success (epitope mapping):** $\text{DockQ}_{\text{selected}}^{B\text{↔}T}\text{≤}0.23$ **vs.** $\text{DockQ}_{\text{selected}}^{B\text{↔}T}\text{>}0.49$ | | | | | | |
| **Format-specific cutoff** | | | | | | |
| **PR-AUC** | Fv | $0.97\text{±}0.03$ | $0.93\text{±}0.08$ | $0.93\text{±}0.09$ | $0.95\text{±}0.06$ | $0.98\text{±}0.03$ |
|  | V_H_H | $0.96\text{±}0.04$ | $1.00\text{±}0.01$ | $0.99\text{±}0.02$ | $0.96\text{±}0.03$ | $0.98\text{±}0.02$ |
| **Best** $\text{ipTM}_{\text{max}}^{B\text{↔}T}$ **cutoff** | Fv | $0.70\text{±}0.15$ | $0.79\text{±}0.07$ | $0.90\text{±}0.06$ | $0.68\text{±}0.11$ | $0.75\text{±}0.06$ |
|  | V_H_H | $0.78\text{±}0.09$ | $0.85\text{±}0.11$ | $0.87\text{±}0.06$ | $0.78\text{±}0.13$ | $0.78\text{±}0.08$ |
| **False discovery rate** | Fv | $7.5\text{±}7.7$% | $7.7\text{±}6.5$% | $5.0\text{±}6.4$% | $10.2\text{±}8.2$% | $8.9\text{±}9.2$% |
|  | V_H_H | $0.0\text{±}5.6$% | $2.6\text{±}4.7$% | $4.2\text{±}5.4$% | $5.4\text{±}7.7$% | $0.0\text{±}2.2$% |
| **False negative rate** | Fv | $9.3\text{±}8.6$% | $3.2\text{±}5.5$% | $8.1\text{±}6.4$% | $3.6\text{±}5.9$% | $4.7\text{±}8.0$% |
|  | V_H_H | $13.5\text{±}10.9$% | $5.0\text{±}7.2$% | $4.2\text{±}9.1$% | $14.6\text{±}12.9$% | $9.3\text{±}8.7$% |
| **Pooled cutoff of Table 3 applied to each format** | | | | | | |
| **Pooled** $\text{ipTM}_{\text{max}}^{B\text{↔}T}$ **cutoff** | Fv, V_H_H | $0.73\text{±}0.10$ | $0.79\text{±}0.05$ | $0.86\text{±}0.05$ | $0.74\text{±}0.09$ | $0.75\text{±}0.06$ |
| **False discovery rate** | Fv | $5.9\text{±}6.9$% | $7.7\text{±}6.4$% | $7.8\text{±}6.6$% | $7.5\text{±}7.6$% | $8.9\text{±}9.0$% |
|  | V_H_H | $3.0\text{±}8.0$% | $4.9\text{±}6.6$% | $4.2\text{±}5.8$% | $5.4\text{±}10.5$% | $0.0\text{±}2.0$% |
| **False negative rate** | Fv | $11.1\text{±}9.4$% | $3.2\text{±}6.4$% | $4.8\text{±}6.2$% | $10.9\text{±}8.3$% | $4.7\text{±}8.5$% |
|  | V_H_H | $13.5\text{±}11.0$% | $2.5\text{±}4.9$% | $4.2\text{±}7.2$% | $14.6\text{±}11.0$% | $9.3\text{±}9.1$% |

**Supplementary Table S2. Pairwise comparison of model responses to antigen mutations.** All models were evaluated on the same 88 complexes, independently of WT eligibility. The 2, 3 and 5 mutation conditions are pooled, with nine individual mutants per complex. Diagonal entries report the percentages below the model-specific high-accuracy ipTM cutoff from **Table 3** in the order shuffled antigen $\text{∣}$ mutant (in italics). Above-diagonal entries report adjusted p-values for differences in mean per-complex antigen-mutant below-cutoff fractions. Below-diagonal entries report adjusted p-values for differences in mean per-complex shuffled-antigen below-cutoff fractions. Two-sided paired label-swap tests keep all variants from the same complex together and use 20,000 permutations with a plus-one correction; rate tests use exact enumeration when there are at most 16 nonzero paired differences. Benjamini-Hochberg correction is applied across the ten model pairs separately for each test and table. Asterisks indicate adjusted $p\text{<}0.05\text{.}$ For each model and target (WT, mutant or shuffle), the prediction with the highest common ranking score, $R\text{,}$ is selected from the 25 candidates generated across all five seeds and five diffusion samples. Results therefore correspond to the full five-seed sampling budget, matching **Figure 4**.

|  | **OpenDDE** | **Protenix-v2** | **AlphaFold 3** | **ESMFold2** | **ESMFold2 fast** |
| --- | --- | --- | --- | --- | --- |
| **OpenDDE** | 93.9% $\text{∣}$ *82.3%* | $\text{<}0.001$^*^ | $\text{<}0.001$^*^ | $\text{<}0.001$^*^ | $\text{<}0.001$^*^ |
| **Protenix-v2** | 0.056 | 97.0% $\text{∣}$ *93.2%* | 0.871 | 0.871 | 0.871 |
| **AlphaFold 3** | 0.390 | 0.378 | 95.5% $\text{∣}$ *91.7%* | 0.871 | 0.871 |
| **ESMFold2** | 0.003^*^ | 0.299 | 0.056 | 98.4% $\text{∣}$ *92.4%* | 1.000 |
| **ESMFold2** **fast** | 0.003^*^ | 0.300 | 0.056 | 1.000 | 98.4% $\text{∣}$ *92.4%* |

**Supplementary Table S3. Pairwise comparison of model median** $\Delta\text{ipTM}$ **responses to mutations and shuffled antigen.** All models were evaluated on the same 88 complexes, independently of WT eligibility. The 2, 3 and 5 mutation conditions are pooled, with nine individual mutants per complex and mutated partner; shuffled antigen comprises five variants per complex. Diagonal entries report median $\Delta\text{ipTM}$ (WT minus mutant or shuffle) in the order shuffled antigen $\text{∣}$ mutant (in italics). Above-diagonal entries report adjusted p-values for differences in median mutant $\Delta\text{ipTM}$ for the partner named in each section. Below-diagonal entries report adjusted p-values for differences in median shuffled-antigen $\Delta\text{ipTM}\text{;}$ these comparisons are repeated in both sections. Two-sided paired label-swap tests keep all variants from the same complex together and use 20,000 permutations with a plus-one correction. Benjamini-Hochberg correction is applied across the ten model pairs separately for antibody mutations, antigen mutations and shuffled antigen. Asterisks indicate adjusted $p\text{<}0.05\text{.}$ For each model and target (WT, mutant or shuffle), the prediction with the highest common ranking score, $R\text{,}$ is selected from the 25 candidates generated across all five seeds and five diffusion samples. Results therefore correspond to the full five-seed sampling budget, matching **Figure 4**.

| **Antibody mutations** | | | | | |
| --- | --- | --- | --- | --- | --- |
|  | **OpenDDE** | **Protenix-v2** | **AlphaFold 3** | **ESMFold2** | **ESMFold2** **fast** |
| **OpenDDE** | 0.17 $\text{∣}$ *0.09* | 0.503 | 0.653 | 0.492 | 0.503 |
| **Protenix-v2** | 0.481 | 0.15 $\text{∣}$ *0.12* | 0.358 | 0.791 | 0.287 |
| **AlphaFold 3** | 0.555 | 0.875 | 0.15 $\text{∣}$ *0.08* | 0.358 | 0.791 |
| **ESMFold2** | 0.007^*^ | 0.021^*^ | 0.141 | 0.27 $\text{∣}$ *0.13* | 0.287 |
| **ESMFold2** **fast** | 0.283 | 0.283 | 0.449 | 0.481 | 0.23 $\text{∣}$ *0.07* |
| **Antigen mutations** | | | | | |
|  | **OpenDDE** | **Protenix-v2** | **AlphaFold 3** | **ESMFold2** | **ESMFold2** **fast** |
| **OpenDDE** | 0.17 $\text{∣}$ *0.08* | 0.776 | 0.776 | 0.419 | 0.776 |
| **Protenix-v2** | 0.481 | 0.15 $\text{∣}$ *0.09* | 0.635 | 0.635 | 0.695 |
| **AlphaFold 3** | 0.555 | 0.875 | 0.15 $\text{∣}$ *0.07* | 0.223 | 0.860 |
| **ESMFold2** | 0.007^*^ | 0.021^*^ | 0.141 | 0.27 $\text{∣}$ *0.13* | 0.223 |
| **ESMFold2** **fast** | 0.283 | 0.283 | 0.449 | 0.481 | 0.23 $\text{∣}$ *0.07* |

**Supplementary Table S4. Model repositories and commits.** Source repository and exact commit hash used to implement each model in this study.

| **Model** | **Repository** | **Commit** |
| --- | --- | --- |
| **ESMFold2** | github.com/Biohub/esm | 26b0bc2b771e3e419ea74f445a5f35cc094a1509 |
| **AlphaFold 3** | github.com/google-deepmind/AlphaFold 3 | 3dfc5a91beee404474d614df1031fbdefaef3277 |
| **Protenix-v2** | github.com/bytedance/Protenix | 3248f8e7e1c94f0b5835a40ebdc649d74d631a82 |
| **OpenDDE** | github.com/aurekaresearch/OpenDDE | 266ce4c49d492ad1077866000d83704999985f46 |

**Supplementary Table S5. Compatible package pins.** Original package version pins for each model, alongside the corresponding pins required for compatibility with our compute specifications (aarch64 CPUs paired with NVIDIA GH200 GPUs running CUDA 12.7). Version strings suffixed with +cu126 indicate CUDA 12.6 builds, used to ensure compatibility with the GPUs.

|  | **ESMFold2** | **Protenix-v2** | **OpenDDE** |
| --- | --- | --- | --- |
| **Original pins** | unpinned | torch==2.7.1 torchvision==0.22.1 torchaudio==2.7.1 triton==3.3.1 | gemmi==0.6.7 torch==2.7.1 torchvision==0.22.1 torchaudio==2.7.1 triton==3.3.1 |
| **Compatible pins** | torch==2.13.0+cu126 triton==3.7.1 | torch==2.10.0+cu126 torchvision==0.25.0+cu126 torchaudio==2.10.0+cu126 triton==3.6.0 | gemmi==0.7.3 torch==2.10.0+cu126 torchvision==0.25.0+cu126 torchaudio==2.10.0+cu126 triton==3.6.0 |

**Supplementary Table S6. Maximum metric variation across identical Protenix-v2 runs.** Maximum absolute difference in ipTM and pTM for a given complex across 3 identical runs, under fixed seeds with deterministic and non-deterministic settings.

| **Complex PDB ID** | $\text{max}\text{\vert}\Delta\text{pTM}\text{\vert}$ **(non-det)** | $\text{max}\text{\vert}\Delta\text{pTM}\text{\vert}$ **(det)** | $\text{max}\text{\vert}\Delta\text{ipTM}\text{\vert}$ **(non-det)** | $\text{max}\text{\vert}\Delta\text{ipTM}\text{\vert}$ **(det)** |
| --- | --- | --- | --- | --- |
| **9lds** | 0.000021 | 0.000000 | 0.000022 | 0.000000 |
| **9rit** | 0.000026 | 0.000000 | 0.000057 | 0.000000 |
| **9hzj** | 0.005892 | 0.000000 | 0.001877 | 0.000000 |
| **9ias** | 0.043868 | 0.000000 | 0.160019 | 0.000000 |
| **9iar** | 0.030269 | 0.000000 | 0.154675 | 0.000000 |
